# Geographic and demographic variability in serum PFAS concentrations for pregnant women in the United States

**DOI:** 10.1038/s41370-023-00520-6

**Published:** 2023-01-25

**Authors:** Nicole M. DeLuca, Kent Thomas, Ashley Mullikin, Rachel Slover, Lindsay W. Stanek, Andrew N. Pilant, Elaine A. Cohen Hubal

**Affiliations:** Center for Public Health and Environmental Assessment, Office of Research and Development, U.S. Environmental Protection Agency, Research Triangle Park, NC USA

## Abstract

**Background:**

While major pathways of human PFAS exposure are thought to be drinking water and diet, other pathways and sources have also been shown to contribute to a person’s cumulative exposure. However, the degree of contribution of these other sources to PFAS body burdens is still not well understood and occurrence data for PFAS in conssumer products and household materials are sparse. Questionnaire data concordant with biomonitoring may improve understanding of associations between other PFAS exposure pathways and exposure in human populations.

**Objective:**

This study aims to better understand maternal and early-life exposures to PFAS from various potential sources and pathways in the context of household and community level characteristics.

**Methods:**

PFAS data from the National Children’s Study (NCS) Vanguard Data and Sample Archive Access System were analyzed from serum of 427 pregnant women residing in 7 counties throughout the United States. Location and self-reported questionnaire responses were used to analyze variability in serum concentrations based on demographics, housing characteristics, behaviors, and geography. Spatial mapping analyses incorporated publicly available data to further hypothesize potential sources of exposure in two NCS counties.

**Results:**

Location was associated with serum concentrations for all PFAS chemicals measured. Questionnaire responses for race/ethnicity, income, education level, number of household members, drinking water source, home age, and fast-food consumption were associated with PFAS levels. Statistical differences were observed between participants with the same questionnaire responses but in different locations. Spatial mapping analyses suggested that participants’ proximity to local point sources can overshadow expected trends with demographic information.

**Significance:**

By increasing understanding of maternal and early-life PFAS exposures from various potential sources and pathways, as well as highlighting the importance of proximity to potential sources in identifying vulnerable populations and locations, this work reveals environmental justice considerations and contributes to risk management strategies that maximize public health protection.

**Impact:**

This work increases understanding of maternal and early-life PFAS exposures, reveals environmental justice considerations, and contributes to study design and risk management strategies.

## Introduction

Per- and polyfluoroalkyl substances (PFAS) are a class of synthetic chemicals that have been widely used in manufacturing since the 1940s due to their water-resistant, stain-resistant, fire-resistant, and anti-stick properties [1]. Due to their extensive use and their propensity to bioaccumulate, they are ubiquitous in the environment and present a risk of adverse health outcomes [1]. The most likely exposure pathways occur through drinking water and diet, but exposures also arise from house dust, air, building materials and furnishings, cleaning products, consumer products, and packaging [2, 3].

Nationally representative biomonitoring conducted in the United States as part of the National Health and Nutrition Examination Survey (NHANES) found four PFAS – perfluorooctanoic acid (PFOA), perfluorooctane sulfonic acid (PFOS), perfluorohexane sulfonic acid (PFHxS), and perfluorononanoic acid (PFNA) – in more than 98% of participants [4]. Following a voluntary phase-out in the early 2000s, serum concentrations of legacy PFAS chemicals in the U.S. general population have mostly decreased over the last 20 years [4–7]. However, newer PFAS chemicals have not followed this trend [3, 5].

Prior studies have identified demographic metrics such as income, race and ethnicity, marital status, and age as significant determinants for PFAS exposure in women in the United States. Higher income is often associated with higher serum levels, which is mainly attributed to consumer product use or dietary differences [4, 8–17]. Inconsistent correlations have been shown between maternal education level and PFAS exposure [12–14, 16]. Race and ethnicity have been found to be significant predictors of exposure with varied findings across different racial and ethnic communities [13, 18–20], including in studies looking at associations with children’s PFAS exposure and maternal race and ethnicity [12, 16]. Marriage status and participant age have sometimes been associated with exposure to some PFAS chemicals [4, 13, 14, 16, 21].

Differences in the home environment and housing characteristics have also been found to correlate with PFAS exposure in U.S. studies. Several studies found higher levels of PFAS in house dust from newer versus older homes, which was thought to be due to increased square footage of PFAS-treated carpeting in newer homes [14, 22, 23]. Knobeloch et al. (2012) found the highest levels of PFOS and PFHxS in house dust samples from homes built between 1968 and 1995 [24]. Differences in PFAS levels in house dust with home age have also been attributed to differences in building construction and materials, as well as how long the materials have been in use [22, 25]. The number of household residents may also provide an indicator of PFAS exposure, with higher PFAS levels found in homes with 1-2 residents compared to larger families [23, 26]. Lower PFAS in homes with more residents was suggested to be due to more frequent cleaning in houses with more residents, resulting in greater removal of surface protective layer from consumer products treated with PFAS [23, 26].

Individual behavior differences, such as drinking water source and diet, have also been shown to influence PFAS exposure levels. While some studies found higher serum PFAS concentrations in people drinking tap water, other studies have detected PFAS in bottled water and found that individuals drinking bottled water as a primary source had higher concentrations of PFAS [14, 27, 28]. Consumption of fried foods (e.g., French fries), fast-food (e.g., burgers, sandwiches), and microwave popcorn has also been shown to be a potentially significant pathway of PFAS exposure, thought to be due to the food’s contact with PFAS-containing wrapping materials [18, 29–32].

Studies focused on cohorts of pregnant women have also found associations between maternal PFAS serum levels and infant cord blood serum levels, indicating that transplacental transfer occurs which underscores the need for further research on PFAS exposures in pregnant women [16]. Several studies have indicated that many of the aforementioned variables still hold predictive value for PFAS serum concentrations in pregnant women. In pregnant women cohort studies, levels of PFAS were associated with demographic factors such as educational attainment, income, age, and race [14, 16, 33–36]. The home environment has also been found to be significant in explaining PFAS exposures in pregnant women, where women living in homes more than 20 years old had lower levels of PFOA and PFNA compared to those living in newer homes [14]. Individual behaviors also remain useful predictors for cohorts of pregnant women. Drinking barreled water was associated with lower PFAS concentrations, while drinking bottled water was associated with higher PFAS concentrations [14, 29]. Diet also related to PFAS concentrations in pregnant women, with greater consumption of dairy milk and cheese indicating higher serum levels of PFNA and perfluorodecanoic acid (PFDA) [13]. In the same study, pregnant women eating fish, poultry, and dairy indicated higher levels of perfluoroundecanoic acid (PFUA), PFDA, PFNA, and PFOS [13].

A recent report from the U.S. Government Accountability Office recommended that U.S. EPA conduct nationwide PFAS exposure studies to “determine the extent which disadvantaged communities are exposed to PFAS in drinking water nationally… [to] help EPA understand whether PFAS in drinking water contributes to the cumulative burden of pollution in disadvantaged communities” [37]. Previously, the National Children’s Study (NCS) Main Study was designed to assess relationships between environmental and other exposures and children’s health in the U.S. in a nationally representative longitudinal birth cohort study. The NCS Initial Vanguard Study (IVS) pilot tested recruitment methods and protocols designed for the NCS Main Study, and it recruited over 1000 pregnant women from seven counties throughout the U.S. in 2009 and 2010. Most women who participated in the IVS pilot had at least one home visit during pregnancy to collect biomonitoring specimens, environmental samples, and questionnaire data. To better understand important household and community factors associated with early-life exposures to PFAS chemicals, this study analyzed data for PFAS measured in serum from 427 of the IVS women along with associated questionnaire data. In addition, publicly available spatial data on demographics, housing characteristics, and PFAS levels in drinking water were explored to understand potential differences in community-level drivers of exposure in two of the IVS counties. The study provides better understanding of maternal and early-life exposures to PFAS chemicals from various potential sources and pathways in the context of household and community level characteristics.

## Methods

### Study population and data collection

Serum and questionnaire data reported in this study were retrieved from the National Children’s Study (NCS) Vanguard Data and Sample Archive Access System in February 2020 just prior to transfer of the NCS Archive to NICHD’s Data and Specimen Hub (DASH) [38]. The NCS Main Study was a proposed nationally representative longitudinal birth cohort study intended to assess relationships between environmental and other exposures and children’s health in the United States [39, 40]. While the full NCS Main Study was never implemented, a pilot study called the Initial Vanguard Study (IVS) was implemented in 2009–2010 to test recruitment methods and protocols for the anticipated Main Study [40–43]. Household recruitment for IVS enrolled over 1000 women that were pregnant or were trying to become pregnant across seven locations in the United States, but this population was not considered to be nationally representative [43–45]. Biomonitoring specimens, environmental samples, and questionnaire data were collected from IVS participants during this pilot study [46, 47].

PFAS concentrations were reported in serum from 427 pregnant women in their third trimester and included measurements of PFOA, PFOS, PFNA, PFHxS, and PFDA. Serum concentrations were analyzed at the U.S. Centers for Disease Control and Prevention (CDC) National Center for Environmental Health using solid-phase extraction high-performance liquid chromatography isotope-dilution tandem mass spectrophotometry [15]. The limit of detection (LOD) for PFOA, PFOS, PFNA, PFHxS, and PFDA were 0.10, 0.20, 0.08, 0.10, and 0.10 ng/mL, respectively. Samples below the LOD were substituted with LOD/$\surd 2$ for summary statistic calculations and subsequent analyses due to a high percent detection (>80%) for these PFAS chemicals.

**Fig. 1:**
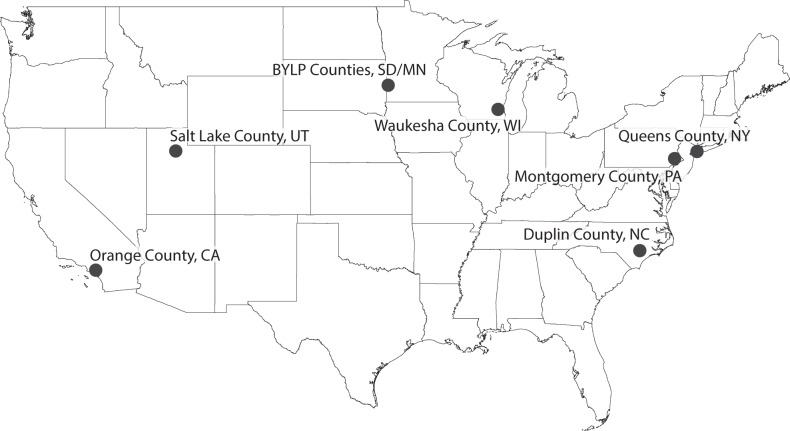
U.S. map showing locations of the 7 NCS IVS counties. Locations in which NCS IVS cohort participants with serum PFAS measurements resided. BYLP is a combination of 4 rural counties - Brookings County, SD, Yellow Medicine County, MN, Lincoln County, MN, and Pipestone County, MN.

The IVS participants with serum PFAS measurements resided in 7 county-level locations (Fig. 1) – Montgomery County, Pennsylvania (*n* = 47), Queens County, New York (*n* = 39), Orange County, California (*n* = 52), Duplin County, North Carolina (*n* = 68), Salt Lake County, Utah (*n* = 94), and Waukesha County, Wisconsin (*n* = 35). One of the seven county-level locations, designated herein as BYLP (*n* = 92), was composed of participants living in four adjacent rural counties – Brookings County, South Dakota, and Yellow Medicine, Pipestone, and Lincoln counties, Minnesota. While this county-level location data was available for the participants in the NCS Vanguard Data and Sample Archive Access System, it is no longer available for this dataset in the NICHD’s DASH database in order to eliminate personally identifiable information.

### Statistical analysis

Summary statistics were calculated for the archived PFAS serum measurement data for IVS participants as a whole, for participants by location, and for participants by questionnaire responses. Summary statistics for participants with similar questionnaire responses but different locations were also calculated. For context, summary statistics for serum PFAS concentrations for pregnant women ages 18 through 50 (*n* = 19) from the 2009–2010 cycle of the National Health and Nutrition Examination Survey (NHANES), a nationally representative population, were also reported in this study [48].

Specific questionnaire variables were selected for this study to gain insights on the study population’s demographics (household income, maternal education, race/ethnicity, marriage status, age), housing characteristics (home age, number of household members), and behaviors (drinking water source, quantity of French fries eaten at one sitting) that have been shown to influence PFAS serum concentrations in previous studies reviewed in the introduction. French fries were used here as a proxy for consumption of fast food, and data on participants’ self-reported quantity of French fries eaten at one sitting was pulled from a food frequency questionnaire administered during the study. To preserve participant confidentiality, frequencies in the summary statistics below 10 were reported as “<10” and some questionnaire response categories with small frequencies were combined for subsequent analyses.

Household annual income responses were combined into two categories—less than $50,000 and $50,000 or more. Responses for marriage status were grouped into three categories—married, not married but living with a partner, or other. Maternal education level responses were combined into two categories—(1) having a high school diploma/GED or less and (2) having attended some college or more. Home age response was categorized into three groups—homes built in 1981 or later, 1961 to 1980, and 1960 and before. Responses for the number of French fries eaten at one sitting was combined into two groups—more than 10/more than ½ cup and less than 10/less than ½ cup. A Pearson’s Chi-squared correlation matrix for the selected survey variables is shown in Fig. S1.

One-way ANOVA analyses were performed for each PFAS chemical using serum and questionnaire data from NICHD’s DASH database in order to investigate associations between participant serum PFAS concentrations, locations, and individual questionnaire responses. Two-way ANOVA analyses for multivariate linear main effects and linear mixed effects models were then used to investigate associations between multiple questionnaire responses and serum PFAS concentrations while accounting for potential confounders and interactions between variables. Reported *F*-values are calculated as the ratio of two variances, the mean square of the independent variables divided by the mean square of the residuals. A larger *F*-value indicates that a variable is more likely to be performing better than by chance in the model. The *p*-value of the *F*-value (Pr(>F)) indicates the likelihood of that calculated *F*-value if the null hypothesis was true in that there was no difference in means.

Pairwise *t*-tests were used to analyze statistically significant differences in the mean between serum concentrations in the seven locations for each chemical and were corrected for multiple comparisons using the Tukey method [49]. Pairwise t-tests were also used to analyze statistically significant differences in the mean between participant serum concentrations from the same questionnaire response groups in different locations (e.g. serum from lower income household participants in Montgomery County versus serum from lower income household participants in Queens County). Statistical significance for these analyses was defined as *p* < 0.05. All statistical analyses in this study were performed in R version 1.3.9 [50].

### Spatial mapping

The spatial mapping performed in ArcMap geographic information systems (GIS) software was used to generate hypotheses about potential sources and explanations for variability in serum PFAS concentrations between locations and questionnaire response groups. Study locations chosen as case studies for spatial analysis were Montgomery County, Pennsylvania and Queens County, New York. These counties were chosen based on the results of the statistical difference tests in serum concentrations and availability of public spatial data on demographics, housing characteristics, and national drinking water sampling campaign results (i.e., sources) within those counties. Public demographic and housing data was retrieved from the decennial U.S. Census and the American Community Survey (ACS) [51]. Yearly median household income data at the zip-code level were downloaded for the 2010 U.S. Census and were subsequently grouped into a lower income range (less than $65,000) and higher income range ($65,000 or more) which reflected the similar lower and higher income ranges used for the NCS questionnaire response analysis. Zip code-level data on years in which occupied homes were built were downloaded from the ACS [51] and subsequently grouped into ranges that most closely reflected the ranges of years that were grouped in the NCS questionnaire responses. The locations of federal sites, including Superfund and military bases where AFFF use was known or suspected, and facilities for industries associated with PFAS use, manufacturing, or release were gathered from U.S. EPA’s Enforcement and Compliance History Online (ECHO) database [52]. Types of industries mapped for this study included furniture and carpets, paints and coatings, textiles and leather, chemical manufacturing, cleaning product manufacturing, consumer products, and electronics. PFAS measurements in drinking water from U.S. EPA’s Third Unregulated Contaminant Monitoring Rule (UCMR3), sampled between 2013 and 2015, were used in spatial mapping at the zip-code level [53]. However, this sampling campaign did not cover all zip codes nationally nor within the NCS counties and the focus of UCMR is generally on large systems that serve >10,000 people and some smaller systems that serve 3000–10,000 people [54].

## Results

### Study population

Population characteristics for NCS participants who both had self-reported questionnaire responses and PFAS serum measurements are found in Table 1. The majority of this population self-identified as non-Hispanic white women who were married. Participant ages ranged from 17 to 43 years old with the mean being 29 years old. The mean number of people living in the household with participants was 4 people. Higher or lower annual household income was fairly evenly distributed in the population. Maternal education level was slightly skewed toward the lower education response. Homes built in 1981 or later housed more participants compared to homes built in 1961 to 1980 and those built in 1960 or before. More participants reported their drinking water source as filtered tap water than tap water or bottled water. A majority of participants reported eating 10 or more (1/2 cup or more) of French fries per sitting.

**Table 1:** Categorical questionnaire derived population characteristics of NCS IVS cohort participants who had serum PFAS measurement data (*n* = 427).

Questionnaire Variable	Responses	Percent of Population
Marriage Status	Married	74%
	Not married but living together with a partner	25%
	Other	<1%
Race/Ethnicity	Hispanic	12%
	Non-Hispanic, Black/African American only	6%
	Non-Hispanic, White only	64%
	Non-Hispanic, Asian only	5%
	Non-Hispanic, NHOPI only	<1%
	Non-Hispanic, multiple races	8%
	Non-Hispanic, missing or other races	4%
Household Income	Less than $50 K	48%
	$50 K or More	45%
Maternal Education Level	High School/GED or Less	56%
	Some College or More	43%
Home Age	1981 or After	34%
	1961–1980	23%
	1960 or Before	22%
Drinking Water Source	Tap Water	36%
	Filtered Tap Water	39%
	Bottled Water	27%
	Other	4%
Number of French Fries Eaten at One Sitting	Less than 10 or Less than 1/2 Cup	22%
	10 or More or 1/2 Cup or More	58%

All participants did not necessarily record a response for every questionnaire variable, and some response categories were combined for statistical analyses and preservation of confidentiality. Numerical survey responses not shown.

### Statistical analysis

**Fig. 2:**
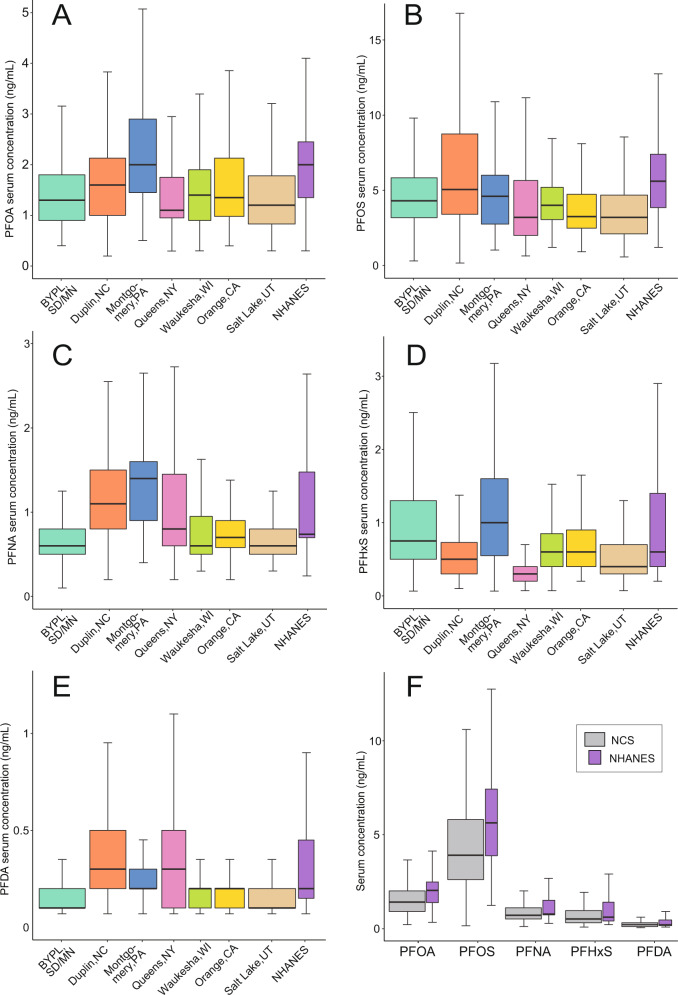
PFAS serum concentrations from NCS IVS cohort by location. Boxplots showing serum concentrations for (**A**) PFOA, (**B**) PFOS, (**C**) PFNA, (**D**) PFHxS, and (**E**) PFDA from NCS IVS participants in each of the 7 county-level locations, as well as serum concentrations from pregnant women in the 2009–2010 cycle of NHANES. (**F**) Serum concentrations from all NCS participants compared to those from NHANES pregnant women for each PFAS chemical. Boxplot widths are proportional to samples sizes for each population.

Serum concentrations of PFAS in the NCS IVS cohort varied between chemicals (Fig. 2). The median concentrations of PFOA, PFOS, PFNA, PFHxS, and PFDA were 1.40 ng/mL, 3.90 ng/mL, 0.70 ng/mL, 0.50 ng/mL, and 0.20 ng/mL, respectively. The median serum concentrations reported for this study population were lower than the median serum concentrations reported for pregnant women in NHANES for the same survey years (Fig. 2) [48]. This was also observed in another cohort of pregnant U.S. women compared to NHANES [13]. Like NHANES, the participants in the NCS cohort had median concentrations of PFOS that were the highest of the measured PFAS chemicals along with the largest ranges in concentrations. Both NCS and NHANES participants had the lowest median concentrations of PFDA. PFOA had the second highest median concentration in serum in both NCS and NHANES, followed by PFNA and PFHxS.

### Geographic variability

IVS participants’ serum concentrations were found to have a statistically significant association with geographic location for all measured PFAS chemicals (*p* < 0.05). Participants in Montgomery County, Pennsylvania had the highest median serum concentrations of PFOA, PFNA and PFHxS; participants in Duplin County, North Carolina had the highest median serum concentrations of PFOS and PFDA (Fig. 2; Table S1). The lowest median serum concentrations of PFOA and PFHxS were observed in participants from Queens County, New York, while the lowest median concentrations of PFDA and PFNA were observed in participants from the combined 4-county location BYLP (Fig. 2; Table S1). Participants in Salt Lake County, Utah had the lowest median serum concentrations of PFOS (Fig. 2; Table S1). Serum concentrations from pregnant women residing in different counties were compared through pairwise tests of mean difference; statistical significance varied by PFAS chemical (Table 2).

**Table 2:** Absolute mean differences (ng/mL) between serum concentrations from pregnant women residing in the 7 NCS IVS counties.

	Duplin	Montgomery	Orange	Queens	Salt Lake	Waukesha
PFOA						
BYLP	0.24	0.80*	0.24	0.12	0.33	0.00
Duplin	—	0.56	0.00	0.12	0.33	0.00
Montgomery	—	—	0.56	0.68*	0.89*	0.56*
Orange	—	—	—	0.12	0.33	0.00
Queens	—	—	—	—	0.21	0.12
Salt Lake	—	—	—	—	—	0.33
PFOS						
BYLP	5.79*	0.48	0.70	0.33	1.09	0.29
Duplin	—	5.31	6.49*	5.46*	6.88*	6.08
Montgomery	—	—	1.18	0.15	1.57	0.77
Orange	—	—	—	1.03	0.39	0.42
Queens	—	—	—	—	1.42	0.62
Salt Lake	—	—	—	—	—	0.81
PFNA						
BYLP	0.54*	0.80*	0.22	0.66*	0.05	0.17
Duplin	—	0.26	0.33*	0.11	0.50*	0.38*
Montgomery	—	—	0.58*	0.14*	0.75*	0.63*
Orange	—	—	—	0.44	0.17	0.05
Queens	—	—	—	—	0.61*	0.49
Salt Lake	—	—	—	—	—	0.12
PFHxS						
BYLP	0.25*	0.27	0.31	0.66*	0.38*	0.33
Duplin	—	0.52*	0.06	0.40*	0.13	0.08
Montgomery	—	—	0.58	0.93*	0.65*	0.60*
Orange	—	—	—	0.34*	0.07	0.02
Queens	—	—	—	—	0.28*	0.32*
Salt Lake	—	—	—	—	—	0.05
PFDA						
BYLP	0.23*	0.12*	0.10*	0.27*	0.02	0.06
Duplin	—	0.11	0.14*	0.04	0.21*	0.17*
Montgomery	—	—	0.03	0.15	0.10*	0.06
Orange	—	—	—	0.17	0.08	0.03
Queens	—	—	—	—	0.25*	0.21*
Salt Lake	—	—	—	—	—	0.04

Statistical significance at *p* < 0.05 from pairwise t-tests between county means, adjusted for multiple comparisons, are indicated with an asterisk.

### Questionnaire responses

Most of the selected survey variables related to demographics were correlated with each other (Fig. S1). Home age was not correlated with any other selected NCS survey variable. Drinking water source was correlated with many demographic variables, and quantity of French fries eaten was only correlated with maternal education level.

Differences in geometric mean for the PFAS serum concentrations between survey response groups are shown in Table 3. For all PFAS except PFDA, geometric mean PFAS serum concentrations were higher for participants that reported higher household incomes. For all PFAS measured, participants with higher maternal education levels had higher geometric mean serum concentrations. Household income and education level were statistically significant at *p* < 0.05 for serum concentrations of PFOA and PFHxS (Table 3). For homes with larger numbers of household members, serum PFAS concentrations generally decreased. Higher geometric mean serum concentrations were generally found in homes built in 1960 or before, while the lowest geometric mean concentrations varied between the two categories of newer homes for all chemicals. Home age was not statistically significant at *p* < 0.05 for serum concentrations of any PFAS, but it was nearly significant for PFNA concentrations (*p* = 0.06). Participants drinking filtered tap water had the highest geometric mean serum concentrations of PFOA, PFNA, and PFHxS, while participants drinking tap water had the highest geometric mean concentration of PFOS (Table 3). The lowest geometric mean serum concentrations were observed for participants drinking bottled water for all chemicals except PFDA, where geometric mean did not differ by response. Drinking water source response was only statistically significant at *p* < 0.05 for PFOA serum concentrations (Table 3). Higher geometric mean serum concentrations were observed for participants eating larger quantities of French fries for all PFAS measured. Higher quantity of French fries eaten at one sitting was significantly associated with higher serum concentrations of all PFAS except PFDA (Table 3). Participant age and marriage status were not significantly associated with serum concentrations for any of the PFAS chemicals.

**Table 3:** Geometric mean (geometric standard deviation) serum concentrations (ng/mL) for selected categorical survey responses from the NCS IVS cohort.

Survey Question	PFOA	PFOS	PFNA	PFHxS	PFDA
Household Income					
Less than $50 K	1.24 (1.83)*	3.82 (2.21)	0.75 (1.83)	0.50 (2.34)*	0.18 (2.15)
$50 K or More	1.57 (1.77)	4.05 (1.76)	0.79 (1.68)	0.68 (2.14)	0.18 (1.89)
Education Level					
High School/GED or Less	1.29 (1.79)*	3.76 (2.21)	0.76 (1.79)	0.51 (2.39)*	0.18 (2.13)
Some College or More	1.49 (1.87)	4.03 (1.77)	0.79 (1.74)	0.64 (2.15)	0.19 (1.95)
Home Age					
Built in 1981 or After	1.41 (1.88)	3.82 (1.78)	0.77 (1.72)	0.55 (2.24)	0.19 (2.01)
Built Between 1961–1980	1.31 (1.80)	3.95 (1.86)	0.70 (1.66)	0.59 (2.18)	0.16 (1.96)
Built in 1960 or Before	1.62 (1.81)	4.26 (1.99)	0.85 (1.90)	0.68 (2.22)	0.19 (2.00)
Drinking Water Source					
Tap Water	1.32 (1.84)*	3.92 (1.93)	0.77 (1.84)	0.54 (2.27)	0.18 (2.11)
Filtered Tap Water	1.53 (1.83)	3.89 (1.86)	0.78 (1.75)	0.63 (2.38)	0.18 (1.95)
Bottled Water	1.23 (1.77)	3.87 (2.42)	0.76 (1.73)	0.50 (2.25)	0.18 (2.16)
Number of French Fries Eaten per Sitting					
Less than 10 or less than 1/2 cup	1.23 (1.87)*	3.37 (2.34)*	0.69 (1.85)*	0.45 (2.52)*	0.17 (2.21)
10 or more or 1/2 cup or more	1.46 (1.84)	4.12 (1.90)	0.80 (1.75)	0.64 (2.27)	0.18 (2.01)

Asterisks indicate results for statistical significance at *p* < 0.05 from one-way analysis of variance (ANOVA) with log PFAS concentrations.

In multivariate main effects and mixed effects models, household income and the number of household members were statistically significant for all PFAS except PFDA (Tables 4,  S2). Household income was not associated with serum PFDA concentrations in these multivariate models. Race/ethnicity was also statistically significant in the main effects and mixed effects models for PFHxS (Tables 4,  S2). Additional variables and variables with interactions that were significant in the mixed effect models are shown in Table S2.

**Table 4:** Multivariate linear main effects model results for associations between selected NCS survey variables and log serum PFAS concentrations (ng/ml), using data from participants that answered all selected survey questions (*n* = 279).

Survey Variables	PFOA		PFOS		PFNA		PFHxS		PFDA	
	F Value	Pr(>F)	F Value	Pr(>F)	F Value	Pr(>F)	F Value	Pr(>F)	F Value	Pr(>F)
Participant Age	1.15	0.285	1.46	0.227	0.10	0.758	1.01	0.315	0.73	0.393
Race/Ethnicity	1.28	0.258	1.20	0.274	0.10	0.747	9.19	0.003*	1.56	0.212
Marriage Status	0.51	0.599	0.23	0.795	0.12	0.885	2.14	0.120	0.08	0.925
Household Income	18.63	<0.001*	7.11	0.008*	10.42	0.001*	17.94	<0.001*	6.11	0.014*
Education Level	0.20	0.652	1.70	0.193	0.49	0.484	1.07	0.301	0.57	0.450
Number of Household Members	20.87	<0.001*	4.20	0.041*	7.94	0.005*	26.96	<0.001*	5.20	0.023*
Home Age	2.28	0.104	0.41	0.665	1.45	0.237	0.90	0.408	1.90	0.152
Drinking Water Source	1.31	0.272	0.19	0.906	0.33	0.803	0.87	0.460	1.48	0.220
Number French Fries per Sitting	1.69	1.94	3.53	0.061	3.49	0.063	1.10	0.296	2.54	0.112
AIC	495.13		507.06		468.08		632.74		576.63	

Asterisks indicate results for statistical significance where *p* < 0.05.

### Questionnaire responses and location

#### Demographics and location

**Fig. 3:**
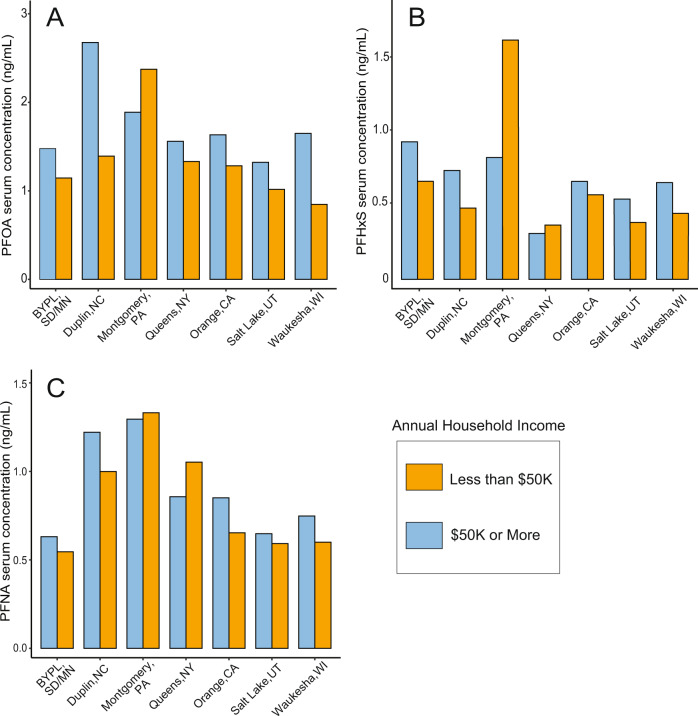
PFAS serum concentrations by income and location. Geometric mean serum concentrations for (**A**) PFOA, (**B**) PFHxS, and (**C**) PFNA from NCS IVS participants from each of the 7 county-level locations, showing participants with household incomes less than $50 K per year (orange) and $50 K or higher per year (blue). Geometric standard deviations can be found in Table S3.

Higher income participants had higher serum concentrations than lower income participants in all counties except for Montgomery County, Pennsylvania (Fig. 3; Table S3). For all counties, participants with higher education levels had higher serum PFOA concentrations than participants with lower education levels in the same county (Table S4). The highest median serum PFOA concentrations were measured in participants from higher income households with higher education levels in Duplin County, North Carolina, while the lowest median serum concentrations were measured in participants from lower income households in Waukesha, Wisconsin and higher education level participants from Salt Lake County, Utah (Tables S3,  S4).

Higher income participants had higher serum concentrations of PFHxS than lower income participants in all counties except for Montgomery County and Queens County (Fig. 3; Table S3). Participants with higher education levels had higher serum PFHxS concentrations than participants with lower education levels except for Montgomery County, PA (Table S4). The highest serum PFHxS concentrations were measured in participants from lower income households with lower education levels in Montgomery County, PA while the lowest PFHxS serum concentrations were measured in participants from higher income households and lower education level participants from Queens County, NY (Tables S3, S4).

Only serum concentrations of PFOA from participants in lower income households in Montgomery County were statistically different from lower income household participants in any of the other counties (Table S5). Results from pairwise t-tests between serum PFAS concentrations for the same income and education response groups in different locations are shown in Table S5 and Table S6. These differences in exposures between the same socioeconomic groups in different counties, particularly between lower socioeconomic status participants in Montgomery County and the other NCS counties, suggests that proximity to point sources could be influencing PFAS exposure for Montgomery County participants more than behaviors or consumer product use that might result from socioeconomic status.

#### Housing characteristics and location

**Fig. 4:**
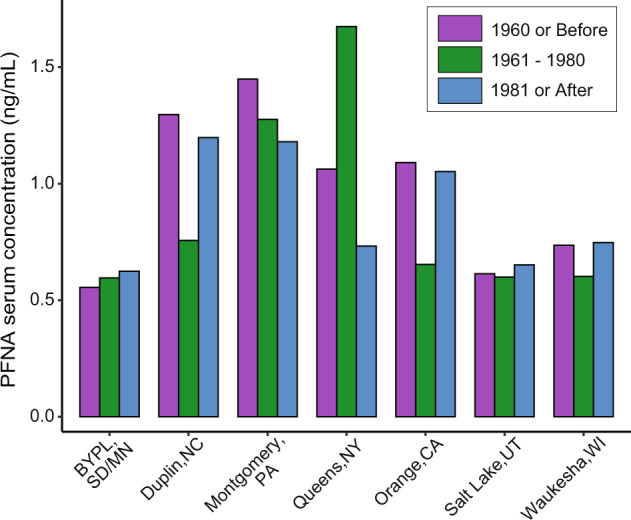
PNA serum concentrations by home age and location. Geometric mean serum concentrations for PFNA for NCS IVS participants from each of the 7 county-level locations, showing participants living in houses built before 1960 (purple), between 1961–1980 (green), and after 1980 (blue). Geometric standard deviations can be found in Table S7.

The highest PFOA serum concentrations were observed in participants living in homes built in 1960 or before in Montgomery County, PA (Table S7). Participants living in homes built in 1981 or after in Queens County, NY had the lowest serum PFOA concentrations of any other home age response group in any NCS county (Table S7). In contrast to the pattern observed in other NCS locations, participants in Queens County that lived in homes built between 1961 and 1980 had the highest serum PFNA concentrations compared to other participants in Queens County living in older or newer homes (Table S7; Fig. 4). This group of Queens County participants also had the highest PFNA serum concentrations of any home age response group from any county (Fig. 4). The lowest PFNA serum concentrations were observed in participants living in homes built in 1960 or before in the BYLP counties (Table S7). While the associations between home age and serum PFAS were not statistically significant (*p* = 0.06), the spatial differences in participant serum concentrations between home age response groups suggest that an environmental factor or proximity to a point source, rather than the age or composition of materials in the homes themselves, could be influencing the trends in PFAS exposure observed in different locations.

#### Behaviors and location

The lowest PFOA serum concentrations were observed in participants that reported drinking bottled water from Waukesha, WI (Table S8). The highest serum PFOA concentrations were observed in participants from Montgomery County, PA, and serum concentrations from these Montgomery County participants were similar no matter their questionnaire response for drinking water source (Table S8). These similar levels in serum irrespective of drinking water source could indicate that the participants were all exposed to PFOA through the same major pathway, likely drinking contaminated tap water. This chemical’s persistence in the body could then continue to influence the participants’ serum PFOA concentrations even after contamination had been identified and interventions to drink bottled water or filtered tap water had been implemented. Statistical differences between drinking water source and PFAS concentrations between locations are found in Table S9. The lack of statistical differences between counties for each drinking water response group suggests that the behavior itself is generally more influential on serum concentrations than spatial differences or point sources between most counties. The exception to this observation is serum concentrations from Montgomery County, PA, in which the statistical differences suggest that a point source of PFAS may have affected drinking water differently than in many of the other NCS counties.

The lowest serum concentrations for PFOA, PFOS, and PFHxS were observed in participants from Queens County, NY who reported eating smaller quantities of French fries per sitting, and the lowest serum concentrations for PFNA were observed in participants from Waukesha County, WI who reported eating smaller quantities of French fries per sitting (Table S10). Interestingly, the highest PFOA and PFHxS serum concentrations were observed in participants from Montgomery County, PA who also reported eating smaller quantities of French fries at one sitting. Smaller quantity eaters in Duplin County, NC also had the highest serum PFOS concentrations. The highest PFNA concentrations were observed in participants from Queens County, NY that reported eating larger quantities of French fries per sitting (Table S10).

### Spatial mapping case studies

To further explore hypotheses that could explain observations from NCS participants’ serum PFAS concentrations, spatial analyses were conducted using GIS. Two GIS case studies are presented to illustrate this exploration. While conclusions cannot be drawn from these spatial mapping analyses, they do provide an opportunity to generate hypotheses that can be tested in future studies.

#### PFOA and income

Lower income participants living in Montgomery County, PA had higher PFOA serum concentrations than the higher income participants from Montgomery County, which is opposite to the effect of income observed in the other NCS counties (Fig. 3). To investigate potential spatial drivers of exposure that could have influenced serum concentrations for these questionnaire response groups, GIS was used to conduct a case study analysis for the county and surrounding area.

**Fig. 5:**
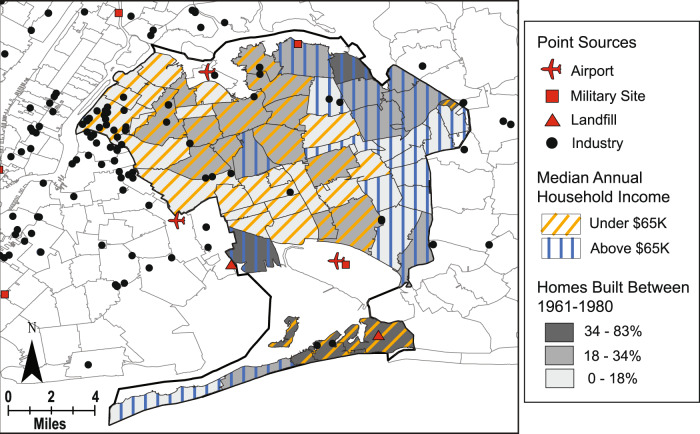
GIS case study in Queens County, New York. Map of Queens County, New York (bold black line) showing percent of housing built between 1961–1980 per zip code (gray shading) from the American Community Survey, median household income per zip code from 2010 U.S. Census where blue slashes indicate higher income and orange slashes indicate lower income, PFAS-related industry facilities (black dots), airports (red planes), and military sites (red squares), and landfills (red triangles).

Lower annual median household income zip codes according to the U.S. Census were generally located in the eastern part of the county. Another larger zip code with lower annual median income was located on the western edge of the county, and several smaller lower income zip codes were scattered throughout the western part of the county (Fig. 5). One of the lower income zip codes in eastern Montgomery County was sampled by UCMR3 and found to have high mean concentrations (75 ppt) of PFOA in its public water supplies. Several zip codes north of the county and adjacent to the lower median income zip codes in Montgomery County were also found to have high mean concentrations of PFOA (91–168 ppt) in their public drinking water supplies. A zip code in eastern Montgomery County which had lower annual median household income and in which high concentrations of PFOA were reported in drinking water contains the site of deactivated Naval Air Station Joint Reserve Base Willow Grove (NASJRB/WG) and active Willow Grove Air Reserve Station (WGARS) in Horsham, PA. This military site was designated as a U.S. EPA Superfund site for other chemicals of concern but is also currently undergoing cleanup efforts to mitigate PFAS-contaminated soil and groundwater from AFFF use [55].

The large zip code in western Montgomery County with lower annual median household income contains an airport, which is another potential contamination of the area due to AFFF use. However, UCMR3 measurements in that zip code’s public water supplies did not yield a detectable level of PFOA. While a larger zip code with lower median household income in south-central Montgomery County did not have detectable PFOA in drinking water according to UCMR3, it is proximal to a cluster of facilities from industries that are known to have had PFAS in use in some capacity. These industries, airports, and military sites could also be contributing occupational exposures to the NCS participants; however, this study did not analyze occupation-related questionnaires that may be available for these participants. From this spatial analysis, a hypothesis could be proposed that the higher median serum concentrations of PFOA observed in Montgomery County participants with lower household incomes were primarily driven by their proximity to contaminated drinking water intakes near military installations rather than factors directly related to socioeconomic status.

#### PFNA, income, and home age

Lower income participants living in Queens County, NY had higher PFNA serum concentrations than higher income participants from Queens County (Fig. 3). This observation was opposite to that in almost all other NCS counties. A different effect of home age on serum PFNA concentrations was exhibited for participants from Queens County compared to the other counties, where the highest serum concentrations were from participants living in homes built between 1961 and 1980 (Fig. 4). To investigate potential spatial drivers of exposure that may have influenced serum concentrations for these questionnaire response groups in Queens County, NY, a case study GIS analysis was conducted for the county and surrounding area.

Many of the zip codes in eastern and central Queens County had lower annual median household incomes, while many of the zip codes in western Queens County had higher annual median household incomes (Fig. 5). Three zip codes with larger percentages of houses built between 1961 and 1980 were located in the south-central region of the county, the north-central region, and the western side of the southern peninsula region in the county. Three airports are adjacent to the lower annual median income zip codes in eastern and southern Queens County, and a cluster of industry facilities are located in northeastern Queens County. The southwestern-most zip code in the peninsula region of the county has a lower median annual household income and a higher percentage of homes built between 1961 and 1980, both of which were associated with higher PFNA serum concentrations in the NCS participants. This zip code is near the John F. Kennedy International Airport and a national defense port of entry, and it also contains a landfill and two other industry facilities. Another landfill is adjacent to a zip code with a higher percentage of homes built between 1961 and 1980, albeit a higher annual income household zip code. Leachate from landfills have been shown to contaminate local surface waters through the release of residual materials from disposed of consumer products [2, 56].

From this spatial analysis, a hypothesis could be proposed that the higher median serum concentrations of PFNA observed in Queens County participants living in lower income households and homes built between 1961–1980 were primarily driven by their proximity to airports and landfills. In addition to environmental contamination of drinking water from AFFF use at airports and landfill leachate, locality of airports could also contribute NCS participants’ serum concentrations through occupational exposure due to a higher likelihood that women may work at a nearby airport.

## Discussion

This study analyzed PFAS serum concentrations from pregnant women in the National Children’s Study Initial Vanguard Study collected throughout 7 locations in the U.S between 2009 and 2010. Geographic location and questionnaire responses were investigated to identify potential drivers of variability in PFAS exposure for 5 chemicals – PFOA, PFOS, PFNA, PFHxS, and PFDA. Measurable levels of PFAS found in the serum of these pregnant women were similar to albeit slightly lower than concentrations from pregnant women reported in NHANES for the same time period. Statistically significant differences were observed in serum concentrations between the 7 locations. Questionnaire responses describing participant demographics, housing characteristics, and behaviors were also associated with serum concentrations, varying by PFAS chemical.

These results agree with previous studies that have shown statistically significant differences in serum concentrations between similar questionnaire factors [4, 8–13, 15, 16, 18, 20–29, 31–33, 57]. By comparing serum concentrations in the NCS counties, particularly between the same questionnaire response groups (e.g., lower income participants in one county versus lower income participants in another county), this study develops new hypotheses about potential drivers or sources of exposure.

### Demographics

Demographics and socioeconomic status, particularly household income and maternal education level, were associated with serum concentrations for several of the PFAS chemicals measured in the NCS cohort. Higher household income and higher maternal education generally indicated higher PFAS serum concentrations. This same pattern was also observed in previous studies, which attributed the variability in serum concentrations with socioeconomic status to the use of different consumer products or differences in diet [4, 8, 9, 11–16, 18, 32, 33, 58]. Higher socioeconomic status could influence decisions about types of products used within the home that can increase occupants’ exposure to PFAS, such as anti-stain carpet treatments [8, 15, 59]. The number of household members living with a participant was also found to be significant in explaining serum concentrations for many of the PFAS measured for the NCS cohort. While other studies have also found associations between PFAS concentrations in homes and the number of people living in the residence, there is not a consensus about the direction of the trend or a potential mechanism [23, 57]. Interestingly, this study did not find the expected association between participant age and serum PFAS concentrations, which has been reported in previous studies. This may be due to the relatively limited age range of the NCS participants, being women of child bearing age, compared to other studies.

Main effects and mixed effects models were used to investigate associations between these questionnaire responses and serum PFAS concentrations while accounting for potential confounders and correlations between survey variables. However, the variable nature of questionnaire data where all questions are not necessarily answered by all participants limits this type of analysis when valuable serum PFAS data with missing survey data must be removed from the models. Additional questionnaire information, such as occupation, could also be collected and analyzed to further investigate the drivers of serum PFAS concentrations observed in the cohort. Univariate analyses also help to inform future study and questionnaire designs.

### Housing characteristics

The age of housing structures was found to be a potentially important predictor for serum PFOA and PFNA concentrations from the pregnant women. Participants living in older homes had the highest serum concentrations, while participants living in homes built between 1961 and 1980 had the lowest serum concentrations. The year in which a residential structure was built has been previously found to influence levels of PFNA and other PFAS in house dust, which is an important indoor exposure pathway for PFAS [14, 22, 24, 25]. However, many of these studies generally found that house dust in older homes had lower levels of PFAS than the dust in newer homes. One study found that newer homes had a significant positive relationship with PFAS concentrations within the home [23]. The variability in house dust concentrations with home age have previously been attributed to the differences in building materials, construction, amount of carpeting, and how long materials had been in use [22, 25]. Given the limited data available and conflicting findings, studies that collect concordant biomonitoring and indoor environmental samples are required to address these gaps in knowledge related to indoor sources and exposures to PFAS.

### Behavior

Drinking water source, reported as either tap water, filtered tap water, or bottled water, was significant in explaining only PFOA serum concentrations. Overall, cohort participants that reported drinking filtered tap water had the highest serum PFOA concentrations and those drinking bottled water had the lowest serum concentrations. While one study also observed higher serum PFOA concentrations in people drinking tap water versus bottled water, another study found higher PFOA concentrations in those drinking bottled water [14, 27]. Geographic variability between serum levels in participants with the same drinking water source responses could be a reflection of the long half-lives of some PFAS chemicals in the body, shown to increase with carbon chain length, which can produce lagged signals of exposure in biomonitoring media that illustrate past exposure rather than current exposure [60, 61]. Thus, the higher serum levels observed in participants drinking filtered water or bottled water in some of the NCS counties could reflect prior PFAS exposure.

The differences observed in serum PFAS concentrations between participants who drank tap water, filtered water, or bottled water could also be a function of their accessibility to in-home water filtering systems and large supplies of bottled water as well as the adequacy of necessary filter maintenance and replacement. In-home water filtering systems and exclusive use of bottled water could therefore be a function of household income. For this NCS cohort, a Pearson’s Chi-squared test was used to determine that the participants’ household income was correlated with their drinking water source at *p* < 0.05. This indicates that the patterns observed in the NCS pregnant women’s serum with drinking water source could ultimately be associated with other exposure sources and pathways related to socioeconomic status.

Questionnaire response for drinking water source alone was not shown to be statistically significant in explaining PFOS nor PFHxS serum concentrations for this cohort. Drinking water contamination from AFFF use for fire extinction at airports, national defense sites, and fire safety training sites has been shown to be a major pathway of PFOS and PFHxS exposure [3, 53, 62–65]. The lack of statistical significance in explaining PFOS serum concentrations suggests that the main driver of PFOS exposure for the NCS pregnant women was likely another factor such as their geographic proximity to sources. The lack of significance of drinking water source for PFOS exposure could also be indicative of the long half-life of this chemical in the body that continues to see a signal from past exposures even if an intervention (e.g., switch from tap water to bottled water) has since occurred. However, it is also important to note that detectable levels of PFAS have been measured in bottled water in addition to tap water [28].

The quantity of French fries eaten per sitting was significantly associated with serum concentrations for all PFAS measured in the NCS women except PFDA. Fast food consumption has previously been shown to have a positive relationship with PFAS serum concentrations, which is thought to be due to the food’s contact with PFAS-containing wrapping materials [18, 29–32]. French fry consumption was not found to be significantly associated with PFDA concentrations for the NCS cohort of pregnant women. While diet is considered to be a major exposure pathway for PFDA, one study showed that food from fast food restaurants in particular was not found to be associated with PFDA which agrees with findings in this study [31, 66]. The associations between serum PFAS concentrations and the quantity of French fries eaten at one time could be better understood in future studies with additional dietary questionnaire information. Here, French fries are used as a proxy for consumption of fast food, but the proportion of fast food in a participant’s diet compared to foods prepared in the home could provide a better causative link for increased serum PFAS concentrations than was possible here.

### Geography

Geographic location was found to be a statistically significant predictor of serum concentrations for all PFAS chemicals measured in the NCS cohort of pregnant women. While investigating potential drivers for differences between serum concentrations in various locations is worthwhile, it can also be useful for exposure investigations to look at potential drivers of statistical similarities. Many of the relative magnitudes and statistical differences, or similarities, in serum concentrations between the 7 counties can be potentially attributed to point sources of contamination located within or near each county.

**Fig. 6:**
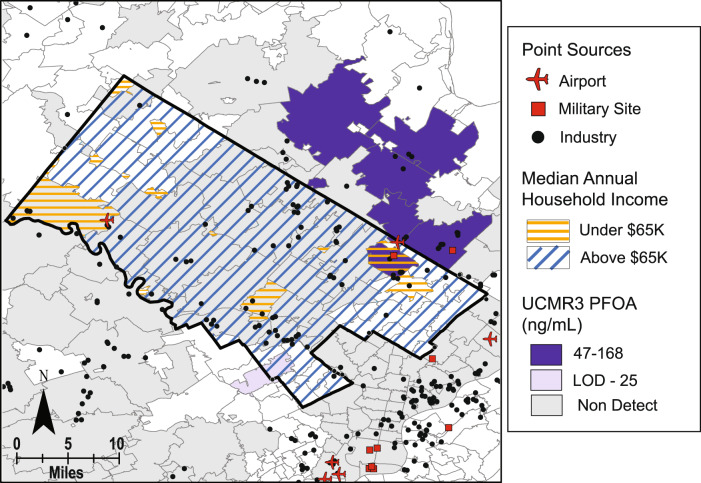
GIS case study in Montgomery County, Pennsylvania. Map of Montgomery County, Pennsylvania (bold black line) showing mean UCMR3 PFOA measurements at the zip code level (purple and gray shading), median household income per zip code from 2010 U.S. Census where blue slashes indicate higher income and orange slashes indicate lower income, PFAS-related industry facilities (black dots), airports (red planes), and military sites (red squares).

**Fig. 7:**
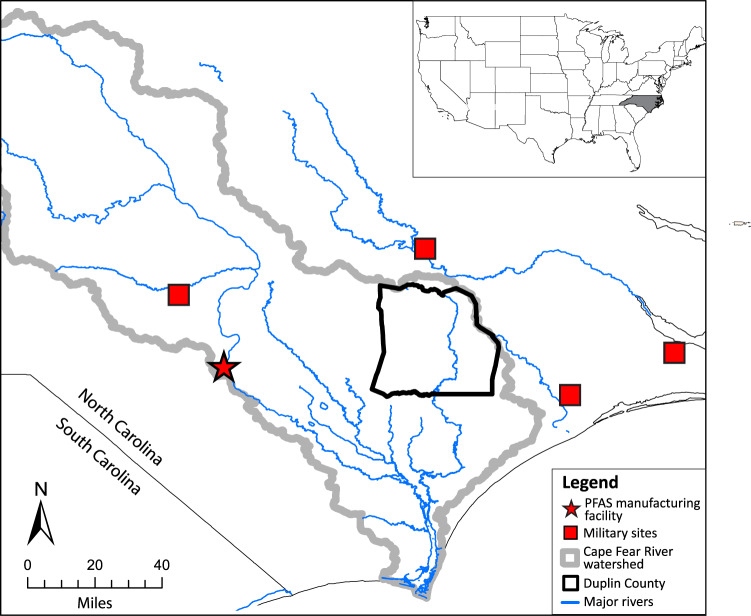
Potential PFAS sources near Duplin County, North Carolina. Map showing the Cape Fear River watershed (gray bold line), Duplin County, North Carolina (black bold line), major rivers around the watershed (blue lines), military sites with known PFAS detections (red squares), and a PFAS manufacturing facility with known PFAS emissions (red star).

Higher median serum concentrations of PFOA, PFNA, and PFHxS from participants residing in Montgomery County, PA, for which PFOA and PFHxS were statistically different than many of the other counties, were likely due to known drinking water contamination from AFFF use at military installations and airports located within and adjacent to the county (Fig. 6) [67]. Duplin County, NC participants had higher median serum concentrations of PFOS, which were statistically different than many of the other counties, which could also be driven by AFFF use at military installations with known PFAS detections located nearby (Fig. 7) [52]. Duplin County, NC is located within the Cape Fear River watershed, which is actively being studied due to findings of PFAS contamination in groundwater and soil that were traced to a local manufacturing facility (Fig. 7) [68, 69]. The contamination of drinking water, as well as exposures from air deposition, from this source could also help to explain the higher median serum concentrations for many PFAS chemicals measured from participants in Duplin County and the statistical differences from many of the other counties. The lack of statistical difference between Montgomery and Duplin Counties for all but one of the PFAS in serum indicates that the types of sources, number of sources, or degrees of exposure from contaminated sites within the two locations could be similar.

The lowest median PFOA, PFOS, and PFHxS serum concentrations were observed in participants from Queens County, NY. In contrast to Montgomery and Duplin Counties, where serum concentrations were the highest of the NCS counties, there are no reported detections of PFAS from military installations within Queens County [52]. However, a large airport is located in the southern part of the county and could be a potential point source of AFFF use (Fig. 5). Serum concentrations of PFOA and PFOS in Queens County were not statistically different than those from most of the other counties, however the low PFHxS concentrations were statistically different than all other counties. This could suggest that some common source of PFHxS in the other NCS counties is not present in Queens County.

Orange County, CA and Salt Lake County, UT had statistically similar serum concentrations for all PFAS chemicals analyzed, and both contain military sites with known PFAS detections [52]. Unlike Montgomery County and Duplin County, there were few statistical differences in serum PFOA and PFOS concentrations between Orange County and Salt Lake County serum levels and those in other NCS counties that also have military installations with known PFAS detections nearby (Waukesha County, BYLP Counties, Queens County) [52]. However, serum concentrations of PFNA, PFHxS, and PFDA were statistically different between many of the NCS counties. The significant differences in serum levels between the counties for these chemicals could be due to differences in consumer products, manufacturing, industrial processes, activities, and dietary preferences that can vary between geographic locations. In contrast, PFOA and PFOS exposures from AFFF use could be considered to be more bifurcated across the country between areas with little or no contamination and highly contaminated areas like those around military installations and airports.

Where previous studies have attributed variability in PFAS exposures to demographic groups’ access to, or choices of, consumer products and household materials, the differences between the same questionnaire response groups in different geographic locations observed in this study suggests that their proximity to local point sources can overshadow the expected trends. Variability in serum concentrations between populations with similar demographics but located in different cities within the U.S. was also observed by Park et al. (2019) [18]. At a minimum, location should be considered a potentially confounding factor in studies determining indicators of exposure.

Furthermore, additional spatial data and finer geographic reporting resolution of biomonitoring data could also strengthen future analyses. The NCS serum data in this study was reported at the county-level, whereas zip-code level geographic information would allow for more targeted analyses of spatial drivers of serum concentrations for particular populations. Including information on drinking water intake locations could also allow for more definitive conclusions about which point sources were contributing to drinking water contamination in different communities.

The two GIS case studies in this work illustrated that the distribution of demographics groups or housing characteristics within a county could be skewed towards closer proximities to potential or known sources of PFAS contamination. Where questionnaire-based studies tend to find that higher socioeconomic status populations have higher serum concentrations of PFAS, this study found that the people living near point sources of PFAS like AFFF use at military installations with higher exposures tend to be populations with lower socioeconomic status. Lower income households and lower education level populations’ proximity to sources could disproportionately contribute to their PFAS exposure through soil and drinking water contamination or through occupational exposure, thereby explaining observed differences in NCS serum concentrations. As the complex linkages between environmental justice and chemical exposures become better understood, the disparities in vulnerable communities’ proximity to sources of contamination should be considered in addition to consumer products, materials in the home, and diet.

### Disclaimer

The views expressed in this article are those of the authors and do not necessarily represent the views or policies of the U.S. Environmental Protection Agency.

### Supplementary information

Supplemental Material

Reporting Checklist

## Data Availability

The data used in this work was retrieved from the National Children’s Study Vanguard Data and Sample Archive and Access System which is now available in NICHD Data and Specimen Hub (DASH). Locational information was removed during the data transfer to DASH.
