# Supplementary material for: Geographic and demographic variability in serum PFAS concentrations for pregnant women in the United States
Source: J Expo Sci Environ Epidemiol. 2023 Jan 25;33(5):710–24. doi: 10.1038/s41370-023-00520-6 (PMC10541323; doi:10.1038/s41370-023-00520-6)
Supplement: Supplementary file 1 — Supplemental Material [file 41370_2023_520_MOESM1_ESM.docx]

Supplemental Material

**Geographic and demographic variability in serum PFAS concentrations for pregnant women in the United States**

Nicole M. DeLuca^1^*, Kent Thomas^1^, Ashley Mullikin^1^, Rachel Slover^1^, Lindsay W. Stanek^1^, Andrew N. Pilant^1^, Elaine A. Cohen Hubal^1^

^1^Center for Public Health and Environmental Assessment, Office of Research and Development, U.S. Environmental Protection Agency, Research Triangle Park, NC, USA

*Corresponding author.

E-mail address: deluca.nikki@epa.gov (N.M. DeLuca)

Address: 109 T.W. Alexander Drive, Research Triangle Park, NC 27709

**Figure S1.** Correlation matrix between selected NCS survey variables. Red squares show correlation where *p*<0.05 from Pearson’s Chi-squared tests.


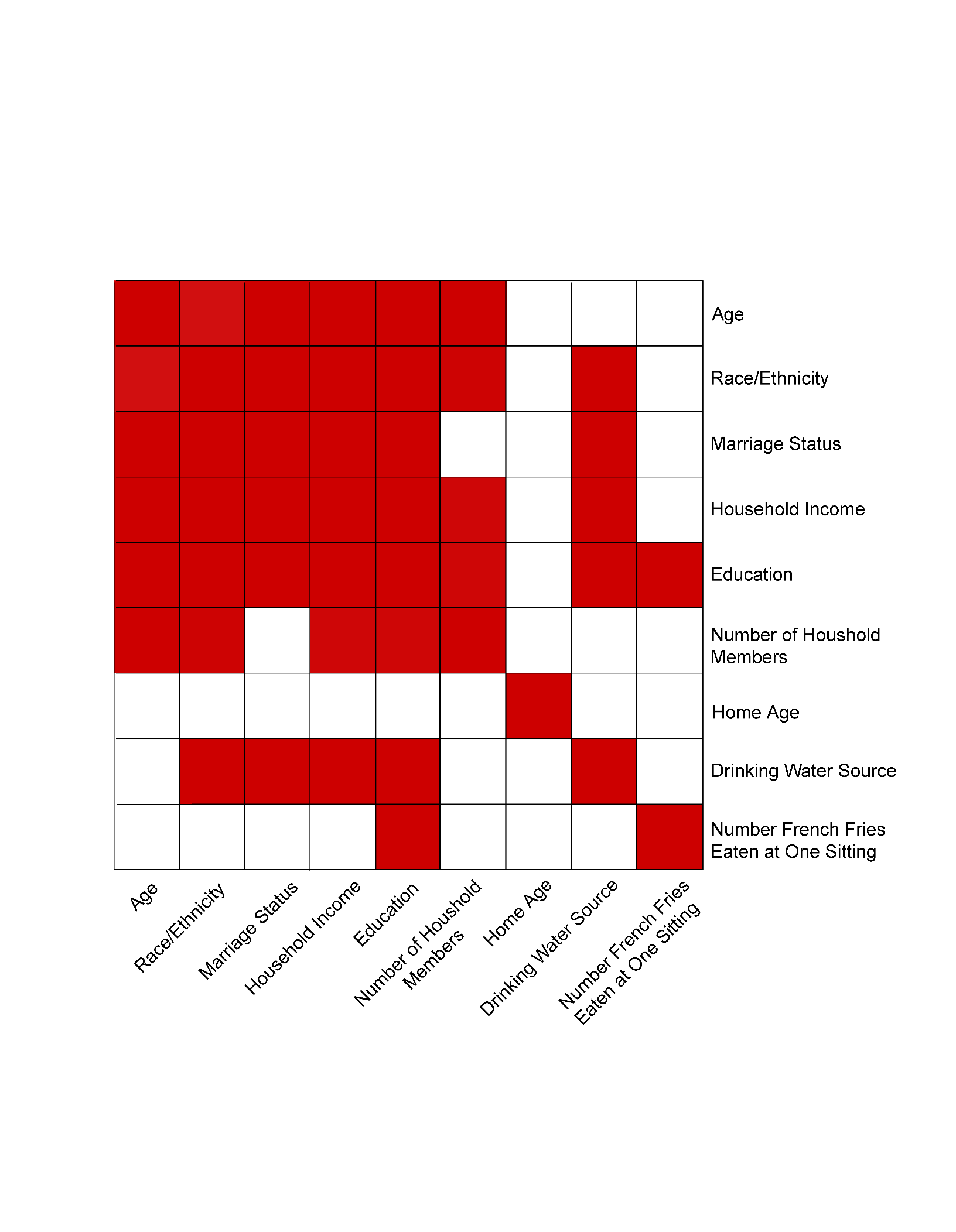


**Table S1.** Summary statistics for serum PFAS measurements (ng/mL) for the NCS IVS cohort (Total) and the 7 NCS IVS counties.

| **PFOA** |  | N | Min | Max | AM | AMSD | GM | GMSD | 10th percentile | 25th percentile | 50th percentile | 75th percentile | 90th percentile | 95th percentile |
| --- | --- | --- | --- | --- | --- | --- | --- | --- | --- | --- | --- | --- | --- | --- |
|  | *Total* | *427* | *0.20* | *7.20* | *1.66* | *1.11* | *1.38* | *1.84* | *0.66* | *0.90* | *1.40* | *2.00* | *3.00* | *3.80* |
|  | Montgomery County, PA | 47 | 0.50 | 6.70 | 2.29 | 1.25 | 1.98 | 1.74 | 1.00 | 1.45 | 2.00 | 2.90 | 4.10 | 4.34 |
|  | New York City (Queens), NY | 39 | 0.30 | 6.90 | 1.61 | 1.38 | 1.24 | 2.05 | 0.40 | 0.95 | 1.10 | 1.75 | 2.86 | 4.34 |
|  | BYLP, SD/MN | 92 | 0.40 | 6.40 | 1.49 | 0.88 | 1.30 | 1.68 | 0.70 | 0.90 | 1.30 | 1.80 | 2.30 | 2.89 |
|  | Orange County, CA | 52 | 0.40 | 6.60 | 1.73 | 1.18 | 1.43 | 1.84 | 0.71 | 0.98 | 1.35 | 2.13 | 3.09 | 3.63 |
|  | Duplin County, NC | 68 | 0.20 | 6.10 | 1.73 | 1.01 | 1.47 | 1.79 | 0.77 | 1.00 | 1.60 | 2.13 | 2.83 | 3.56 |
|  | Salt Lake County, UT | 94 | 0.30 | 4.90 | 1.40 | 0.86 | 1.19 | 1.77 | 0.60 | 0.83 | 1.20 | 1.78 | 2.40 | 3.11 |
|  | Waukesha County, WI | 35 | 0.30 | 7.20 | 1.73 | 1.46 | 1.33 | 2.07 | 0.50 | 0.90 | 1.40 | 1.90 | 2.88 | 4.57 |
|  |  |  |  |  |  |  |  |  |  |  |  |  |  |  |
| **PFOS** |  |  |  |  |  |  |  |  |  |  |  |  |  |  |
|  | *Total* | *427* | *0.14* | *208.00* | *5.32* | *10.87* | *3.86* | *2.02* | *1.60* | *2.60* | *3.90* | *5.80* | *8.70* | *11.38* |
|  | Montgomery County, PA | 47 | 1.00 | 15.90 | 5.15 | 3.36 | 4.18 | 1.97 | 1.78 | 2.75 | 4.60 | 6.00 | 9.46 | 11.99 |
|  | New York City (Queens), NY | 39 | 0.60 | 27.10 | 4.99 | 5.31 | 3.48 | 2.27 | 1.40 | 2.00 | 3.20 | 5.65 | 8.98 | 15.11 |
|  | BYLP, SD/MN | 92 | 0.30 | 12.70 | 4.67 | 2.34 | 4.03 | 1.82 | 1.92 | 3.18 | 4.30 | 5.83 | 7.48 | 9.50 |
|  | Orange County, CA | 52 | 0.90 | 13.60 | 3.96 | 2.56 | 3.32 | 1.83 | 1.41 | 2.48 | 3.25 | 4.73 | 7.29 | 8.86 |
|  | Duplin County, NC | 68 | 0.14 | 208.00 | 10.46 | 25.95 | 5.62 | 2.57 | 2.10 | 3.40 | 5.05 | 8.75 | 13.29 | 16.31 |
|  | Salt Lake County, UT | 94 | 0.60 | 13.80 | 3.57 | 2.04 | 3.09 | 1.73 | 1.53 | 2.10 | 3.20 | 4.68 | 6.07 | 6.70 |
|  | Waukesha County, WI | 35 | 1.20 | 11.10 | 4.38 | 2.34 | 3.82 | 1.73 | 1.68 | 3.05 | 4.00 | 5.20 | 6.62 | 9.92 |
|  |  |  |  |  |  |  |  |  |  |  |  |  |  |  |
| **PFNA** |  |  |  |  |  |  |  |  |  |  |  |  |  |  |
|  | *Total* | *427* | *0.10* | *6.80* | *0.92* | *0.66* | *0.77* | *1.77* | *0.40* | *0.50* | *0.70* | *1.10* | *1.60* | *2.00* |
|  | Montgomery County, PA | 47 | 0.40 | 3.60 | 1.43 | 0.64 | 1.30 | 1.56 | 0.76 | 0.90 | 1.40 | 1.60 | 2.30 | 2.57 |
|  | New York City (Queens), NY | 39 | 0.20 | 6.80 | 1.29 | 1.32 | 0.94 | 2.12 | 0.40 | 0.60 | 0.80 | 1.45 | 2.42 | 3.73 |
|  | BYLP, SD/MN | 92 | 0.10 | 1.60 | 0.63 | 0.25 | 0.59 | 1.52 | 0.40 | 0.50 | 0.60 | 0.80 | 0.99 | 1.00 |
|  | Orange County, CA | 52 | 0.20 | 3.90 | 0.85 | 0.60 | 0.72 | 1.74 | 0.40 | 0.58 | 0.70 | 0.90 | 1.57 | 1.84 |
|  | Duplin County, NC | 68 | 0.20 | 2.80 | 1.18 | 0.56 | 1.04 | 1.68 | 0.50 | 0.80 | 1.10 | 1.50 | 1.90 | 2.13 |
|  | Salt Lake County, UT | 94 | 0.30 | 1.60 | 0.68 | 0.30 | 0.62 | 1.53 | 0.33 | 0.50 | 0.60 | 0.80 | 1.20 | 1.30 |
|  | Waukesha County, WI | 35 | 0.30 | 2.90 | 0.80 | 0.56 | 0.69 | 1.67 | 0.40 | 0.50 | 0.60 | 0.95 | 1.10 | 1.65 |
|  |  |  |  |  |  |  |  |  |  |  |  |  |  |  |
| **PFHxS** |  |  |  |  |  |  |  |  |  |  |  |  |  |  |
|  | *Total* | *427* | *0.07* | *12.60* | *0.81* | *0.94* | *0.56* | *2.30* | *0.20* | *0.30* | *0.50* | *0.95* | *1.70* | *2.27* |
|  | Montgomery County, PA | 47 | 0.07 | 4.90 | 1.30 | 1.08 | 0.94 | 2.42 | 0.40 | 0.55 | 1.00 | 1.60 | 2.66 | 3.45 |
|  | New York City (Queens), NY | 39 | 0.07 | 1.40 | 0.37 | 0.32 | 0.28 | 2.15 | 0.10 | 0.20 | 0.30 | 0.40 | 0.82 | 0.94 |
|  | BYLP, SD/MN | 92 | 0.07 | 5.30 | 1.03 | 0.86 | 0.77 | 2.20 | 0.30 | 0.50 | 0.75 | 1.30 | 2.17 | 2.44 |
|  | Orange County, CA | 52 | 0.20 | 2.40 | 0.72 | 0.49 | 0.61 | 1.76 | 0.30 | 0.40 | 0.60 | 0.90 | 1.28 | 1.74 |
|  | Duplin County, NC | 68 | 0.10 | 12.60 | 0.78 | 1.52 | 0.50 | 2.18 | 0.20 | 0.30 | 0.50 | 0.73 | 1.23 | 1.83 |
|  | Salt Lake County, UT | 94 | 0.07 | 4.50 | 0.65 | 0.75 | 0.45 | 2.24 | 0.20 | 0.30 | 0.40 | 0.70 | 1.20 | 2.11 |
|  | Waukesha County, WI | 35 | 0.07 | 2.30 | 0.70 | 0.49 | 0.56 | 1.97 | 0.30 | 0.40 | 0.60 | 0.85 | 1.06 | 1.86 |
|  |  |  |  |  |  |  |  |  |  |  |  |  |  |  |
| **PFDA** |  |  |  |  |  |  |  |  |  |  |  |  |  |  |
|  | *Total* | *427* | *0.07* | *2.60* | *0.24* | *0.25* | *0.18* | *2.05* | *0.07* | *0.10* | *0.20* | *0.30* | *0.50* | *0.67* |
|  | Montgomery County, PA | 47 | 0.07 | 0.80 | 0.27 | 0.15 | 0.24 | 1.71 | 0.10 | 0.20 | 0.20 | 0.30 | 0.40 | 0.47 |
|  | New York City (Queens), NY | 39 | 0.07 | 2.50 | 0.42 | 0.47 | 0.27 | 2.58 | 0.07 | 0.10 | 0.30 | 0.50 | 0.82 | 1.06 |
|  | BYLP, SD/MN | 92 | 0.07 | 0.80 | 0.15 | 0.10 | 0.13 | 1.67 | 0.07 | 0.10 | 0.10 | 0.20 | 0.20 | 0.30 |
|  | Orange County, CA | 52 | 0.07 | 1.20 | 0.24 | 0.23 | 0.19 | 1.91 | 0.07 | 0.10 | 0.20 | 0.20 | 0.39 | 0.65 |
|  | Duplin County, NC | 68 | 0.07 | 2.60 | 0.38 | 0.35 | 0.29 | 2.12 | 0.10 | 0.20 | 0.30 | 0.50 | 0.63 | 0.87 |
|  | Salt Lake County, UT | 94 | 0.07 | 0.60 | 0.17 | 0.11 | 0.14 | 1.80 | 0.07 | 0.10 | 0.10 | 0.20 | 0.30 | 0.40 |
|  | Waukesha County, WI | 35 | 0.07 | 1.00 | 0.21 | 0.20 | 0.16 | 1.98 | 0.07 | 0.10 | 0.20 | 0.20 | 0.30 | 0.55 |

**Table S2.** Multivariate linear mixed effects model results for associations between selected NCS survey variables and log serum PFAS concentrations (ng/ml), using data from participants that answered all selected survey questions (n = 279). Asterisks indicate results for statistical significance where p < 0.05.

|  | **PFOA** | |  | | **PFOS** | |  | | **PFNA** | | | **PFHxS** | |  | **PFDA** | |  | |  |
| --- | --- | --- | --- | --- | --- | --- | --- | --- | --- | --- | --- | --- | --- | --- | --- | --- | --- | --- | --- |
| **Survey Variables** | F Value | Pr(>F) | | | F Value | Pr(>F) | | | F Value | Pr(>F) | | F Value | Pr(>F) | | F Value | Pr(>F) | | |  |
| Participant Age | 1.19 | 0.276 | | | 1.56 | 0.213 | | | 0.10 | 0.751 | | 1.02 | 0.314 | | 0.78 | 0.378 | | |  |
| Race/Ethnicity | 1.34 | 0.249 | | | 1.28 | 0.259 | | | 0.11 | 0.739 | | 9.24 | 0.003* | | 1.67 | 0.198 | | |  |
| Marriage Status | 2.85 | 0.060 | | | 0.54 | 0.582 | | | 1.11 | 0.331 | | 3.00 | 0.052 | | 0.64 | 0.526 | | |  |
| Household Income | 12.05 | <0.001* | | | 4.61 | 0.033* | | | 7.21 | 0.008* | | 11.75 | <0.001* | | 3.65 | 0.057 | | |  |
| Education Level | 1.41 | 0.237 | | | 3.76 | 0.054 | | | 1.76 | 0.186 | | 3.81 | 0.052 | | 1.21 | 0.272 | | |  |
| Number of Household Members | 20.52 | <0.001* | | | 4.44 | 0.036* | | | 8.47 | 0.004* | | 26.48 | <0.001* | | 5.60 | 0.019* | | |  |
| Home Age | 2.19 | 0.114 | | | 0.31 | 0.735 | | | 1.33 | 0.266 | | 0.70 | 0.500 | | 1.86 | 0.159 | | |  |
| Drinking Water Source | 2.31 | 0.077 | | | 0.34 | 0.795 | | | 0.65 | 0.585 | | 1.74 | 0.159 | | 2.10 | 0.101 | | |  |
| Number of French Fries Eaten per Sitting | 1.95 | 0.164 | | | 4.05 | 0.045* | | | 3.93 | 0.048* | | 1.38 | 0.242 | | 2.55 | 0.112 | | |  |
| Racial Ethnicity*Drinking Water Source | 4.63 | 0.011* | | | 8.57 | <0.001* | | | 2.10 | 0.124 | | 2.62 | 0.075 | | 2.25 | 0.107 | | |  |
| Racial Ethnicity*Education Level | 6.06 | 0.014* | | | 1.48 | 0.225 | | | 8.28 | 0.004* | | 1.33 | 0.251 | | 5.89 | 0.016* | | |  |
| Racial Ethnicity*Household Income | 0.39 | 0.535 | | | 0.26 | 0.607 | | | 0.00 | 0.983 | | 0.04 | 0.848 | | 0.19 | 0.662 | | |  |
| Education Level*Marriage Status | 0.22 | 0.806 | | | 0.43 | 0.650 | | | 0.07 | 0.937 | | 2.44 | 0.089 | | 0.34 | 0.713 | | |  |
| Racial Ethnicity*Number Household Members | 0.16 | 0.689 | | | 0.54 | 0.464 | | | 0.00 | 0.959 | | 1.75 | 0.187 | | 3.68 | 0.056 | | |  |
| Household Income*Marriage Status | 0.48 | 0.489 | | | 0.00 | 0.993 | | | 0.01 | 0.946 | | 0.07 | 0.785 | | 0.33 | 0.567 | | |  |
| Drinking Water Source*Household Income | 0.14 | 0.936 | | | 0.37 | 0.778 | | | 0.63 | 0.595 | | 0.69 | 0.558 | | 0.66 | 0.576 | | |  |
| Household Income*Number Household Members | 0.40 | 0.526 | | | 0.16 | 0.688 | | | 0.99 | 0.320 | | 0.04 | 0.843 | | 0.18 | 0.668 | | |  |
| Education Level*Household Income | 0.80 | 0.372 | | | 0.00 | 0.976 | | | 1.23 | 0.267 | | 0.00 | 0.967 | | 1.97 | 0.161 | | |  |
| Education Level*French Fries | 0.14 | 0.709 | | | 1.85 | 0.176 | | | 0.26 | 0.610 | | 0.04 | 0.842 | | 0.29 | 0.589 | | |  |
| Drinking Water Source*Education Level | 1.96 | 0.120 | | | 2.53 | 0.058 | | | 4.14 | 0.007* | | 0.45 | 0.718 | | 4.29 | 0.006* | | |  |
| Household Income*French Fries | 0.02 | 0.885 | | | 2.13 | 0.146 | | | 0.01 | 0.918 | | 0.76 | 0.385 | | 0.01 | 0.943 | | |  |
| Drinking Water Source*Marriage Status | 2.35 | 0.073 | | | 1.76 | 0.156 | | | 2.97 | 0.032* | | 1.61 | 0.187 | | 1.96 | 0.121 | | |  |
| AIC | 503.24 | | |  | 508.32 | | |  | 469.34 | |  | 650.25 | |  | 577.79 | | |  | |

**Table S3.** Summary statistics for serum PFAS measurements (ng/mL) for the NCS IVS cohort (Total) and the 7 NCS IVS counties, subdivided by self-reported household income level. Total number of responses for each response category is censored to preserve participant confidentiality.

|  |  | N | Min | Max | AM | SD | GM | GMSD |
| --- | --- | --- | --- | --- | --- | --- | --- | --- |
| **PFOA** |  |  |  |  |  |  |  |  |
| Less than $50k | *Total* | - | *0.20* | *6.90* | *1.49* | *0.97* | *1.24* | *1.83* |
|  | Montgomery County, PA | 10 | 1.00 | 4.10 | 2.58 | 1.04 | 2.37 | 1.57 |
|  | New York City (Queens), NY | 25 | 0.30 | 6.90 | 1.80 | 1.60 | 1.33 | 2.20 |
|  | BYLP, SD/MN | 46 | 0.40 | 4.00 | 1.31 | 0.78 | 1.15 | 1.66 |
|  | Orange County, CA | 20 | 0.40 | 3.90 | 1.48 | 0.85 | 1.28 | 1.72 |
|  | Duplin County, NC | 53 | 0.20 | 4.20 | 1.59 | 0.83 | 1.39 | 1.73 |
|  | Salt Lake County, UT | 40 | 0.30 | 3.30 | 1.19 | 0.70 | 1.02 | 1.79 |
|  | Waukesha County, WI | <10 | 0.30 | 2.20 | 1.00 | 0.60 | 0.85 | 1.87 |
| $50k or More | *Total* | - | *0.40* | *7.20* | *1.85* | *1.17* | *1.57* | *1.77* |
|  | Montgomery County, PA | 37 | 0.50 | 6.70 | 2.21 | 1.30 | 1.89 | 1.78 |
|  | New York City (Queens), NY | <10 | 1.10 | 3.50 | 1.78 | 1.16 | 1.56 | 1.73 |
|  | BYLP, SD/MN | 46 | 0.40 | 6.40 | 1.67 | 0.94 | 1.48 | 1.64 |
|  | Orange County, CA | 23 | 0.50 | 6.60 | 1.98 | 1.35 | 1.63 | 1.89 |
|  | Duplin County, NC | <10 | 1.60 | 3.70 | 2.78 | 0.80 | 2.68 | 1.38 |
|  | Salt Lake County, UT | 52 | 0.40 | 4.30 | 1.51 | 0.83 | 1.32 | 1.68 |
|  | Waukesha County, WI | 25 | 0.50 | 7.20 | 2.05 | 1.59 | 1.65 | 1.93 |
|  |  |  |  |  |  |  |  |  |
| **PFOS** |  |  |  |  |  |  |  |  |
| Less than $50k | *Total* | - | *0.14* | *208.00* | *6.06* | *15.48* | *3.82* | *2.21* |
|  | Montgomery County, PA | 10 | 1.50 | 15.90 | 7.10 | 4.36 | 5.78 | 2.07 |
|  | New York City (Queens), NY | 25 | 1.40 | 27.10 | 6.29 | 6.15 | 4.56 | 2.16 |
|  | BYLP, SD/MN | 46 | 0.30 | 12.70 | 4.23 | 2.27 | 3.59 | 1.92 |
|  | Orange County, CA | 20 | 1.20 | 8.50 | 3.31 | 1.72 | 2.92 | 1.69 |
|  | Duplin County, NC | 53 | 0.14 | 208.00 | 11.15 | 29.34 | 5.27 | 2.78 |
|  | Salt Lake County, UT | 40 | 0.60 | 7.40 | 3.08 | 1.64 | 2.66 | 1.77 |
|  | Waukesha County, WI | <10 | 1.20 | 5.00 | 3.03 | 1.21 | 2.80 | 1.57 |
| $50k or More | *Total* | - | *0.90* | *13.80* | *4.71* | *2.59* | *4.05* | *1.76* |
|  | Montgomery County, PA | 37 | 1.00 | 12.70 | 4.62 | 2.89 | 3.83 | 1.91 |
|  | New York City (Queens), NY | <10 | 1.70 | 4.00 | 2.88 | 0.97 | 2.74 | 1.44 |
|  | BYLP, SD/MN | 46 | 0.90 | 10.40 | 5.10 | 2.35 | 4.53 | 1.70 |
|  | Orange County, CA | 23 | 1.00 | 13.60 | 5.08 | 3.11 | 4.25 | 1.87 |
|  | Duplin County, NC | <10 | 3.80 | 10.20 | 7.56 | 2.65 | 7.13 | 1.49 |
|  | Salt Lake County, UT | 52 | 1.10 | 13.80 | 3.98 | 2.26 | 3.48 | 1.68 |
|  | Waukesha County, WI | 25 | 1.40 | 11.10 | 4.99 | 2.41 | 4.48 | 1.63 |
|  |  |  |  |  |  |  |  |  |
| **PFNA** |  |  |  |  |  |  |  |  |
| Less than $50k | *Total* | - | *0.10* | *6.80* | *0.92* | *0.75* | *0.75* | *1.83* |
|  | Montgomery County, PA | 10 | 0.80 | 2.30 | 1.41 | 0.52 | 1.33 | 1.42 |
|  | New York City (Queens), NY | 25 | 0.40 | 6.80 | 1.50 | 1.57 | 1.05 | 2.20 |
|  | BYLP, SD/MN | 46 | 0.10 | 1.40 | 0.59 | 0.23 | 0.55 | 1.54 |
|  | Orange County, CA | 20 | 0.20 | 2.10 | 0.76 | 0.46 | 0.65 | 1.73 |
|  | Duplin County, NC | 53 | 0.20 | 2.80 | 1.14 | 0.58 | 1.00 | 1.73 |
|  | Salt Lake County, UT | 40 | 0.30 | 1.50 | 0.66 | 0.33 | 0.59 | 1.61 |
|  | Waukesha County, WI | <10 | 0.30 | 1.20 | 0.66 | 0.30 | 0.60 | 1.56 |
| $50k or More | *Total* | - | *0.20* | *3.90* | *0.92* | *0.57* | *0.79* | *1.68* |
|  | Montgomery County, PA | 37 | 0.40 | 3.60 | 1.44 | 0.67 | 1.30 | 1.61 |
|  | New York City (Queens), NY | <10 | 0.50 | 2.20 | 1.03 | 0.79 | 0.86 | 1.91 |
|  | BYLP, SD/MN | 46 | 0.20 | 1.60 | 0.68 | 0.26 | 0.63 | 1.49 |
|  | Orange County, CA | 23 | 0.40 | 3.90 | 0.98 | 0.71 | 0.85 | 1.65 |
|  | Duplin County, NC | <10 | 0.50 | 1.90 | 1.34 | 0.54 | 1.22 | 1.70 |
|  | Salt Lake County, UT | 52 | 0.30 | 1.60 | 0.70 | 0.28 | 0.65 | 1.46 |
|  | Waukesha County, WI | 25 | 0.40 | 2.90 | 0.87 | 0.62 | 0.75 | 1.67 |
|  |  |  |  |  |  |  |  |  |
| **PFHxS** |  |  |  |  |  |  |  |  |
| Less than $50k | *Total* | - | *0.07* | *12.60* | *0.76* | *1.11* | *0.50* | *2.34* |
|  | Montgomery County, PA | 10 | 0.40 | 4.90 | 2.23 | 1.68 | 1.62 | 2.48 |
|  | New York City (Queens), NY | 25 | 0.07 | 1.40 | 0.45 | 0.35 | 0.35 | 2.09 |
|  | BYLP, SD/MN | 46 | 0.07 | 4.00 | 0.89 | 0.75 | 0.65 | 2.35 |
|  | Orange County, CA | 20 | 0.20 | 2.40 | 0.66 | 0.47 | 0.56 | 1.74 |
|  | Duplin County, NC | 53 | 0.10 | 12.60 | 0.77 | 1.70 | 0.47 | 2.19 |
|  | Salt Lake County, UT | 40 | 0.07 | 2.60 | 0.53 | 0.53 | 0.37 | 2.35 |
|  | Waukesha County, WI | <10 | 0.20 | 0.80 | 0.48 | 0.23 | 0.43 | 1.65 |
| $50k or More | *Total* | - | *0.07* | *5.30* | *0.91* | *0.78* | *0.68* | *2.14* |
|  | Montgomery County, PA | 37 | 0.07 | 3.10 | 1.05 | 0.69 | 0.81 | 2.29 |
|  | New York City (Queens), NY | <10 | 0.20 | 0.90 | 0.38 | 0.35 | 0.29 | 2.12 |
|  | BYLP, SD/MN | 46 | 0.20 | 5.30 | 1.17 | 0.94 | 0.92 | 1.99 |
|  | Orange County, CA | 23 | 0.20 | 2.40 | 0.79 | 0.56 | 0.65 | 1.89 |
|  | Duplin County, NC | <10 | 0.40 | 1.30 | 0.78 | 0.34 | 0.72 | 1.55 |
|  | Salt Lake County, UT | 52 | 0.20 | 4.50 | 0.75 | 0.88 | 0.53 | 2.13 |
|  | Waukesha County, WI | 25 | 0.07 | 2.30 | 0.79 | 0.54 | 0.64 | 2.03 |
|  |  |  |  |  |  |  |  |  |
| **PFDA** |  |  |  |  |  |  |  |  |
| Less than $50k | *Total* | - | *0.07* | *2.60* | *0.25* | *0.31* | *0.18* | *2.15* |
|  | Montgomery County, PA | 10 | 0.10 | 0.40 | 0.22 | 0.12 | 0.19 | 1.81 |
|  | New York City (Queens), NY | 25 | 0.07 | 2.50 | 0.50 | 0.55 | 0.31 | 2.65 |
|  | BYLP, SD/MN | 46 | 0.07 | 0.40 | 0.13 | 0.08 | 0.12 | 1.65 |
|  | Orange County, CA | 20 | 0.07 | 0.40 | 0.19 | 0.08 | 0.17 | 1.57 |
|  | Duplin County, NC | 53 | 0.07 | 2.60 | 0.36 | 0.38 | 0.27 | 2.14 |
|  | Salt Lake County, UT | 40 | 0.07 | 0.60 | 0.15 | 0.11 | 0.12 | 1.80 |
|  | Waukesha County, WI | <10 | 0.07 | 0.30 | 0.13 | 0.08 | 0.12 | 1.71 |
| $50k or More | *Total* | - | *0.07* | *1.20* | *0.23* | *0.18* | *0.18* | *1.89* |
|  | Montgomery County, PA | 37 | 0.07 | 0.80 | 0.28 | 0.15 | 0.25 | 1.67 |
|  | New York City (Queens), NY | <10 | 0.07 | 0.50 | 0.24 | 0.20 | 0.18 | 2.50 |
|  | BYLP, SD/MN | 46 | 0.07 | 0.80 | 0.16 | 0.12 | 0.14 | 1.67 |
|  | Orange County, CA | 23 | 0.07 | 1.20 | 0.31 | 0.30 | 0.23 | 2.00 |
|  | Duplin County, NC | <10 | 0.07 | 0.60 | 0.41 | 0.20 | 0.34 | 2.42 |
|  | Salt Lake County, UT | 52 | 0.07 | 0.50 | 0.18 | 0.11 | 0.15 | 1.79 |
|  | Waukesha County, WI | 25 | 0.07 | 1.00 | 0.24 | 0.23 | 0.19 | 1.99 |

**Table S4.** Summary statistics for serum PFAS measurements (ng/mL) for the NCS IVS cohort (Total) and the 7 NCS IVS counties, subdivided by self-reported maternal education level. Total number of responses for each response category is censored to preserve participant confidentiality.

|  |  | N | Min | Max | AM | SD | GM | GMSD |
| --- | --- | --- | --- | --- | --- | --- | --- | --- |
| **PFOA** |  |  |  |  |  |  |  |  |
| High School/ GED or Less | *Total* | - | *0.20* | *7.20* | *1.53* | *0.95* | *1.29* | *1.79* |
|  | Montgomery County, PA | 13 | 0.50 | 4.40 | 2.11 | 1.17 | 1.78 | 1.90 |
|  | New York City (Queens), NY | 28 | 0.30 | 5.60 | 1.49 | 1.12 | 1.18 | 2.03 |
|  | BYLP, SD/MN | 41 | 0.50 | 3.70 | 1.35 | 0.66 | 1.22 | 1.56 |
|  | Orange County, CA | 31 | 0.40 | 4.70 | 1.53 | 0.98 | 1.30 | 1.76 |
|  | Duplin County, NC | 61 | 0.20 | 4.20 | 1.59 | 0.82 | 1.39 | 1.73 |
|  | Salt Lake County, UT | 50 | 0.30 | 4.30 | 1.41 | 0.85 | 1.19 | 1.81 |
|  | Waukesha County, WI | 17 | 0.40 | 7.20 | 1.70 | 1.57 | 1.31 | 2.04 |
| Some College, Associate's Degree or More | *Total* | - | *0.30* | *6.90* | *1.82* | *1.25* | *1.49* | *1.87* |
|  | Montgomery County, PA | 34 | 0.70 | 6.70 | 2.36 | 1.29 | 2.06 | 1.68 |
|  | New York City (Queens), NY | 10 | 0.40 | 6.90 | 1.96 | 2.02 | 1.40 | 2.23 |
|  | BYLP, SD/MN | 51 | 0.40 | 6.40 | 1.60 | 1.01 | 1.37 | 1.76 |
|  | Orange County, CA | 20 | 0.50 | 6.60 | 2.07 | 1.41 | 1.70 | 1.92 |
|  | Duplin County, NC | <10 | 1.00 | 6.10 | 2.93 | 1.67 | 2.55 | 1.79 |
|  | Salt Lake County, UT | 42 | 0.40 | 3.60 | 1.33 | 0.71 | 1.16 | 1.69 |
|  | Waukesha County, WI | 18 | 0.30 | 5.90 | 1.77 | 1.40 | 1.36 | 2.13 |
|  |  |  |  |  |  |  |  |  |
|  |  |  |  |  |  |  |  |  |
| **PFOS** |  |  |  |  |  |  |  |  |
| High School/ GED or Less | *Total* | - | *0.14* | *208.00* | *5.82* | *14.26* | *3.76* | *2.21* |
|  | Montgomery County, PA | 13 | 1.00 | 15.90 | 5.26 | 4.01 | 4.03 | 2.21 |
|  | New York City (Queens), NY | 28 | 0.60 | 27.10 | 4.83 | 5.65 | 3.22 | 2.38 |
|  | BYLP, SD/MN | 41 | 0.30 | 12.70 | 4.77 | 2.57 | 4.00 | 1.98 |
|  | Orange County, CA | 31 | 0.90 | 8.50 | 3.16 | 1.69 | 2.75 | 1.73 |
|  | Duplin County, NC | 61 | 0.14 | 208.00 | 10.77 | 27.36 | 5.53 | 2.64 |
|  | Salt Lake County, UT | 50 | 0.90 | 13.80 | 3.61 | 2.47 | 2.98 | 1.86 |
|  | Waukesha County, WI | 17 | 1.20 | 9.80 | 4.03 | 2.07 | 3.53 | 1.75 |
| Some College, Associate's Degree or More | *Total* | - | *0.60* | *15.20* | *4.72* | *2.79* | *4.03* | *1.77* |
|  | Montgomery County, PA | 34 | 1.00 | 12.70 | 5.10 | 3.15 | 4.24 | 1.90 |
|  | New York City (Queens), NY | 10 | 1.60 | 14.70 | 5.71 | 4.71 | 4.47 | 2.02 |
|  | BYLP, SD/MN | 51 | 0.90 | 10.30 | 4.58 | 2.16 | 4.06 | 1.71 |
|  | Orange County, CA | 20 | 1.50 | 13.60 | 5.32 | 3.15 | 4.55 | 1.77 |
|  | Duplin County, NC | <10 | 2.00 | 15.20 | 7.71 | 4.51 | 6.43 | 2.02 |
|  | Salt Lake County, UT | 42 | 0.60 | 7.10 | 3.56 | 1.45 | 3.25 | 1.60 |
|  | Waukesha County, WI | 18 | 1.40 | 11.10 | 4.71 | 2.59 | 4.12 | 1.72 |
|  |  |  |  |  |  |  |  |  |
|  |  |  |  |  |  |  |  |  |
| **PFNA** |  |  |  |  |  |  |  |  |
| High School/ GED or Less | *Total* | - | *0.10* | *6.80* | *0.91* | *0.71* | *0.76* | *1.79* |
|  | Montgomery County, PA | 13 | 0.40 | 2.50 | 1.23 | 0.60 | 1.10 | 1.65 |
|  | New York City (Queens), NY | 28 | 0.20 | 6.80 | 1.41 | 1.53 | 0.95 | 2.32 |
|  | BYLP, SD/MN | 41 | 0.10 | 1.40 | 0.62 | 0.24 | 0.57 | 1.55 |
|  | Orange County, CA | 31 | 0.20 | 2.00 | 0.71 | 0.38 | 0.63 | 1.67 |
|  | Duplin County, NC | 61 | 0.20 | 2.80 | 1.16 | 0.57 | 1.03 | 1.69 |
|  | Salt Lake County, UT | 50 | 0.30 | 1.50 | 0.68 | 0.30 | 0.62 | 1.53 |
|  | Waukesha County, WI | 17 | 0.30 | 1.20 | 0.69 | 0.28 | 0.64 | 1.50 |
| Some College, Associate's Degree or More | *Total* | - | *0.20* | *3.90* | *0.93* | *0.60* | *0.79* | *1.74* |
|  | Montgomery County, PA | 34 | 0.60 | 3.60 | 1.51 | 0.64 | 1.39 | 1.52 |
|  | New York City (Queens), NY | 10 | 0.40 | 1.90 | 0.95 | 0.47 | 0.86 | 1.60 |
|  | BYLP, SD/MN | 51 | 0.20 | 1.60 | 0.65 | 0.26 | 0.60 | 1.50 |
|  | Orange County, CA | 20 | 0.40 | 3.90 | 1.09 | 0.79 | 0.93 | 1.72 |
|  | Duplin County, NC | <10 | 0.40 | 1.80 | 1.30 | 0.45 | 1.19 | 1.66 |
|  | Salt Lake County, UT | 42 | 0.30 | 1.60 | 0.68 | 0.31 | 0.62 | 1.53 |
|  | Waukesha County, WI | 18 | 0.30 | 2.90 | 0.90 | 0.73 | 0.73 | 1.83 |
|  |  |  |  |  |  |  |  |  |
|  |  |  |  |  |  |  |  |  |
| **PFHxS** |  |  |  |  |  |  |  |  |
| High School/ GED or Less | *Total* | - | *0.07* | *12.60* | *0.78* | *1.10* | *0.51* | *2.39* |
|  | Montgomery County, PA | 13 | 0.40 | 4.90 | 1.82 | 1.52 | 1.34 | 2.27 |
|  | New York City (Queens), NY | 28 | 0.07 | 1.40 | 0.38 | 0.36 | 0.26 | 2.34 |
|  | BYLP, SD/MN | 41 | 0.07 | 5.30 | 1.11 | 1.06 | 0.76 | 2.55 |
|  | Orange County, CA | 31 | 0.20 | 2.40 | 0.65 | 0.43 | 0.56 | 1.70 |
|  | Duplin County, NC | 61 | 0.10 | 12.60 | 0.77 | 1.60 | 0.48 | 2.19 |
|  | Salt Lake County, UT | 50 | 0.07 | 3.90 | 0.63 | 0.73 | 0.42 | 2.35 |
|  | Waukesha County, WI | 17 | 0.20 | 1.80 | 0.62 | 0.40 | 0.53 | 1.76 |
| Some College, Associate's Degree or More | *Total* | - | *0.07* | *4.50* | *0.85* | *0.70* | *0.64* | *2.15* |
|  | Montgomery County, PA | 34 | 0.07 | 3.60 | 1.10 | 0.79 | 0.82 | 2.41 |
|  | New York City (Queens), NY | 10 | 0.20 | 0.90 | 0.38 | 0.22 | 0.34 | 1.64 |
|  | BYLP, SD/MN | 51 | 0.20 | 3.50 | 0.96 | 0.66 | 0.79 | 1.93 |
|  | Orange County, CA | 20 | 0.30 | 2.40 | 0.84 | 0.57 | 0.69 | 1.85 |
|  | Duplin County, NC | <10 | 0.30 | 1.90 | 0.83 | 0.60 | 0.66 | 2.07 |
|  | Salt Lake County, UT | 42 | 0.07 | 4.50 | 0.68 | 0.79 | 0.48 | 2.15 |
|  | Waukesha County, WI | 18 | 0.07 | 2.30 | 0.77 | 0.56 | 0.60 | 2.18 |
|  |  |  |  |  |  |  |  |  |
|  |  |  |  |  |  |  |  |  |
| **PFDA** |  |  |  |  |  |  |  |  |
| High School/ GED or Less | *Total* | - | *0.07* | *2.60* | *0.25* | *0.29* | *0.18* | *2.13* |
|  | Montgomery County, PA | 13 | 0.07 | 0.70 | 0.23 | 0.18 | 0.18 | 2.02 |
|  | New York City (Queens), NY | 28 | 0.07 | 2.50 | 0.43 | 0.54 | 0.25 | 2.82 |
|  | BYLP, SD/MN | 41 | 0.07 | 0.40 | 0.13 | 0.07 | 0.11 | 1.57 |
|  | Orange County, CA | 31 | 0.07 | 0.70 | 0.20 | 0.13 | 0.17 | 1.78 |
|  | Duplin County, NC | 61 | 0.07 | 2.60 | 0.38 | 0.36 | 0.28 | 2.12 |
|  | Salt Lake County, UT | 50 | 0.07 | 0.50 | 0.16 | 0.10 | 0.14 | 1.76 |
|  | Waukesha County, WI | 17 | 0.07 | 0.40 | 0.16 | 0.10 | 0.14 | 1.78 |
| Some College, Associate's Degree or More | *Total* | - | *0.07* | *1.20* | *0.24* | *0.20* | *0.19* | *1.95* |
|  | Montgomery County, PA | 34 | 0.10 | 0.80 | 0.29 | 0.13 | 0.26 | 1.53 |
|  | New York City (Queens), NY | 10 | 0.07 | 1.00 | 0.37 | 0.26 | 0.30 | 2.05 |
|  | BYLP, SD/MN | 51 | 0.07 | 0.80 | 0.17 | 0.12 | 0.14 | 1.72 |
|  | Orange County, CA | 20 | 0.07 | 1.20 | 0.33 | 0.32 | 0.25 | 1.99 |
|  | Duplin County, NC | <10 | 0.07 | 0.70 | 0.41 | 0.21 | 0.34 | 2.15 |
|  | Salt Lake County, UT | 42 | 0.07 | 0.60 | 0.17 | 0.13 | 0.14 | 1.87 |
|  | Waukesha County, WI | 18 | 0.07 | 1.00 | 0.26 | 0.26 | 0.19 | 2.14 |

**Table S5.** P-values for pairwise t-tests between serum concentrations for the same household income response groups in each NCS IVS county, adjusted for multiple comparisons. Asterisk indicates statistical significance at *p*<0.05.

| *Lower Household Income* | |  |  | | | |  |
| --- | --- | --- | --- | --- | --- | --- | --- |
| **PFOA** | Duplin | Montgomery | Orange | Queens | Salt Lake | Waukesha |  |
| BYLP | 0.6343 | 0.0072* | 0.9904 | 0.9427 | 0.9632 | 0.7852 |  |
| Duplin | - | 0.1124 | 0.9982 | 0.9999 | 0.1335 | 0.2123 |  |
| Montgomery | - | - | 0.0943 | 0.1129 | 0.0010* | 0.0028* |  |
| Orange | - | - | - | 1.0000 | 0.7631 | 0.5585 |  |
| Queens | - | - | - | - | 0.5318 | 0.4127 |  |
| Salt Lake | - | - | - | - | - | 0.9787 |  |
|  |  |  |  |  |  |  |  |
| **PFHxS** | Duplin | Montgomery | Orange | Queens | Salt Lake | Waukesha |  |
| BYLP | 0.3598 | 0.0180* | 0.9907 | 0.0284* | 0.0165* | 0.7762 |  |
| Duplin | - | 0.0002* | 0.9773 | 0.7371 | 0.7775 | 0.9999 |  |
| Montgomery | - | - | 0.0102* | <.0001* | <.0001* | 0.0057* |  |
| Orange | - | - | - | 0.4309 | 0.4614 | 0.9820 |  |
| Queens | - | - | - | - | 1.0000 | 0.9938 |  |
| Salt Lake | - | - | - | - | - | 0.9983 |  |

*Higher Household Income*

| **PFOA** | Duplin | Montgomery | Orange | Queens | Salt Lake | Waukesha |
| --- | --- | --- | --- | --- | --- | --- |
| BYLP | 0.2656 | 0.4220 | 0.9924 | 1.0000 | 0.9560 | 0.9856 |
| Duplin | - | 0.8435 | 0.5489 | 0.7759 | 0.1021 | 0.5643 |
| Montgomery |  | - | 0.9568 | 0.9949 | 0.0505 | 0.9652 |
| Orange |  |  | - | 1.0000 | 0.7367 | 1.0000 |
| Queens |  |  |  | - | 0.9975 | 1.0000 |
| Salt Lake |  |  |  |  | - | 0.6637 |
|  |  |  |  |  |  |  |
| **PFHxS** | Duplin | Montgomery | Orange | Queens | Salt Lake | Waukesha |
| BYLP | 0.9924 | 0.9873 | 0.4989 | 0.0447* | 0.0040* | 0.4202 |
| Duplin | - | 0.9999 | 0.9999 | 0.5122 | 0.9679 | 0.9999 |
| Montgomery | - | - | 0.9075 | 0.1124 | 0.0905 | 0.8702 |
| Orange | - | - | - | 0.4039 | 0.9163 | 1.0000 |
| Queens | - | - | - | - | 0.7055 | 0.4163 |
| Salt Lake | - | - | - | - | - | 0.9280 |

**Table S6.** P-values for pairwise t-tests between serum concentrations for the same maternal education level response groups in each NCS IVS county, adjusted for multiple comparisons. Asterisk indicates statistical significance at *p*<0.05.

*Lower Education Level*

| **PFOA** | Duplin | Montgomery | Orange | Queens | Salt Lake | Waukesha |
| --- | --- | --- | --- | --- | --- | --- |
| BYLP | 0.9325 | 0.3853 | 0.9990 | 1.0000 | 1.0000 | 0.9996 |
| Duplin | - | 0.7898 | 0.9992 | 0.8911 | 0.8345 | 0.9998 |
| Montgomery |  | - | 0.6654 | 0.3476 | 0.2924 | 0.7773 |
| Orange |  |  | - | 0.9945 | 0.9944 | 1.0000 |
| Queens |  |  |  | - | 1.0000 | 0.9974 |
| Salt Lake |  |  |  |  | - | 0.9978 |
|  |  |  |  |  |  |  |
| **PFHxS** | Duplin | Montgomery | Orange | Queens | Salt Lake | Waukesha |
| BYLP | 0.0813 | 0.2719 | 0.6725 | <.0001* | 0.0117* | 0.7314 |
| Duplin | - | 0.0008* | 0.9833 | 0.0166* | 0.9788 | 0.9993 |
| Montgomery |  | - | 0.0171* | <.0001* | 0.0001* | 0.0312* |
| Orange |  |  | - | 0.0067* | 0.7459 | 1.0000 |
| Queens |  |  |  | - | 0.1494 | 0.0634 |
| Salt Lake |  |  |  |  | - | 0.9478 |

*Higher Education Level*

| **PFOA** | Duplin | Montgomery | Orange | Queens | Salt Lake | Waukesha |
| --- | --- | --- | --- | --- | --- | --- |
| BYLP | 0.1377 | 0.0349* | 0.8136 | 1.0000 | 0.8362 | 1.0000 |
| Duplin | - | 0.9792 | 0.7181 | 0.4002 | 0.0245* | 0.2174 |
| Montgomery |  | - | 0.9098 | 0.5498 | 0.0009* | 0.1991 |
| Orange |  |  | - | 0.9813 | 0.2230 | 0.9066 |
| Queens |  |  |  | - | 0.9713 | 1.0000 |
| Salt Lake |  |  |  |  | - | 0.9669 |
|  |  |  |  |  |  |  |
| **PFHxS** | Duplin | Montgomery | Orange | Queens | Salt Lake | Waukesha |
| BYLP | 0.9974 | 1.0000 | 0.9944 | 0.0174* | 0.0294* | 0.8212 |
| Duplin | - | 0.9924 | 1.0000 | 0.5005 | 0.9413 | 0.9999 |
| Montgomery |  | - | 0.9813 | 0.0155* | 0.0338* | 0.7528 |
| Orange |  |  | - | 0.1548 | 0.5587 | 0.9964 |
| Queens |  |  |  | - | 0.7981 | 0.4301 |
| Salt Lake |  |  |  |  | - | 0.9492 |

**Table S7.** Summary statistics for serum PFAS measurements (ng/mL) for the NCS IVS cohort (Total) and the 7 NCS IVS counties, subdivided by self-reported home age. Total number of responses for each response category is censored to preserve participant confidentiality.

|  |  | N | Min | Max | AM | SD | GM | GMSD |
| --- | --- | --- | --- | --- | --- | --- | --- | --- |
| **PFOA** |  |  |  |  |  |  |  |  |
| Built in 1981 or After | *Total* | - | *0.30* | *6.70* | *1.73* | *1.25* | *1.41* | *1.88* |
|  | Montgomery County, PA | <10 | 0.50 | 6.70 | 2.34 | 1.96 | 1.76 | 2.26 |
|  | New York City (Queens), NY | 11 | 0.40 | 2.50 | 1.19 | 0.57 | 1.07 | 1.64 |
|  | BYLP, SD/MN | 32 | 0.40 | 6.40 | 1.62 | 1.17 | 1.37 | 1.73 |
|  | Orange County, CA | 11 | 1.20 | 6.60 | 2.48 | 1.53 | 2.19 | 1.63 |
|  | Duplin County, NC | 18 | 0.50 | 6.10 | 2.12 | 1.47 | 1.69 | 2.02 |
|  | Salt Lake County, UT | 42 | 0.30 | 4.30 | 1.47 | 0.95 | 1.22 | 1.85 |
|  | Waukesha County, WI | 22 | 0.30 | 5.90 | 1.73 | 1.26 | 1.41 | 1.94 |
| Built Between 1961-1980 | *Total* | - | *0.40* | *7.20* | *1.56* | *1.09* | *1.31* | *1.80* |
|  | Montgomery County, PA | 11 | 1.00 | 3.40 | 2.00 | 0.79 | 1.86 | 1.49 |
|  | New York City (Queens), NY | <10 | 1.00 | 6.90 | 3.10 | 3.30 | 2.13 | 2.81 |
|  | BYLP, SD/MN | 21 | 0.50 | 3.00 | 1.48 | 0.68 | 1.32 | 1.64 |
|  | Orange County, CA | 15 | 0.40 | 3.40 | 1.44 | 0.94 | 1.18 | 1.94 |
|  | Duplin County, NC | 11 | 0.80 | 2.60 | 1.49 | 0.64 | 1.37 | 1.55 |
|  | Salt Lake County, UT | 31 | 0.40 | 2.90 | 1.27 | 0.65 | 1.12 | 1.67 |
|  | Waukesha County, WI | <10 | 0.50 | 7.20 | 2.40 | 2.83 | 1.40 | 3.16 |
| Built in 1960 or Before | *Total* | - | *0.40* | *5.60* | *1.91* | *1.09* | *1.62* | *1.81* |
|  | Montgomery County, PA | 20 | 0.70 | 4.60 | 2.61 | 1.22 | 2.29 | 1.73 |
|  | New York City (Queens), NY | 13 | 0.40 | 5.60 | 2.03 | 1.56 | 1.54 | 2.25 |
|  | BYLP, SD/MN | 30 | 0.40 | 3.40 | 1.49 | 0.68 | 1.33 | 1.64 |
|  | Orange County, CA | <10 | 1.10 | 3.90 | 2.50 | 1.98 | 2.07 | 2.45 |
|  | Duplin County, NC | <10 | 1.30 | 3.70 | 2.27 | 0.90 | 2.11 | 1.49 |
|  | Salt Lake County, UT | 13 | 0.40 | 2.30 | 1.39 | 0.63 | 1.24 | 1.73 |
|  | Waukesha County, WI | <10 | 0.90 | 2.50 | 1.74 | 0.63 | 1.64 | 1.50 |
|  |  |  |  |  |  |  |  |  |
|  |  |  |  |  |  |  |  |  |
| **PFOS** |  |  |  |  |  |  |  |  |
| Built in 1981 or After | *Total* | - | *0.90* | *15.20* | *4.50* | *2.74* | *3.82* | *1.78* |
|  | Montgomery County, PA | <10 | 1.00 | 12.70 | 4.91 | 4.10 | 3.53 | 2.45 |
|  | New York City (Queens), NY | 11 | 1.40 | 7.10 | 3.53 | 1.70 | 3.16 | 1.65 |
|  | BYLP, SD/MN | 32 | 0.90 | 12.70 | 4.77 | 2.37 | 4.22 | 1.69 |
|  | Orange County, CA | 11 | 2.40 | 13.60 | 5.42 | 3.59 | 4.58 | 1.80 |
|  | Duplin County, NC | 18 | 1.80 | 15.20 | 5.93 | 3.53 | 5.08 | 1.77 |
|  | Salt Lake County, UT | 42 | 1.10 | 13.80 | 3.77 | 2.39 | 3.20 | 1.77 |
|  | Waukesha County, WI | 22 | 1.40 | 10.20 | 4.19 | 1.93 | 3.81 | 1.58 |
| Built Between 1961-1980 | *Total* | - | *1.00* | *69.50* | *5.11* | *7.07* | *3.95* | *1.86* |
|  | Montgomery County, PA | 11 | 1.90 | 12.20 | 5.20 | 2.98 | 4.56 | 1.70 |
|  | New York City (Queens), NY | <10 | 5.90 | 14.10 | 9.10 | 4.39 | 8.47 | 1.58 |
|  | BYLP, SD/MN | 21 | 2.40 | 10.40 | 5.11 | 2.07 | 4.79 | 1.43 |
|  | Orange County, CA | 15 | 1.00 | 10.40 | 4.13 | 2.75 | 3.30 | 2.06 |
|  | Duplin County, NC | 11 | 1.70 | 69.50 | 10.09 | 19.81 | 4.78 | 2.79 |
|  | Salt Lake County, UT | 31 | 1.40 | 9.60 | 3.42 | 1.72 | 3.08 | 1.58 |
|  | Waukesha County, WI | <10 | 1.80 | 9.80 | 5.02 | 3.08 | 4.29 | 1.90 |
| Built in 1960 or Before | *Total* | - | *0.30* | *18.80* | *5.20* | *3.20* | *4.26* | *1.99* |
|  | Montgomery County, PA | 20 | 1.00 | 11.50 | 5.08 | 2.81 | 4.27 | 1.91 |
|  | New York City (Queens), NY | 13 | 1.40 | 18.80 | 5.92 | 5.19 | 4.45 | 2.15 |
|  | BYLP, SD/MN | 30 | 0.30 | 10.30 | 4.61 | 2.47 | 3.81 | 2.06 |
|  | Orange County, CA | <10 | 3.20 | 3.90 | 3.55 | 0.50 | 3.53 | 1.15 |
|  | Duplin County, NC | <10 | 3.70 | 12.90 | 8.19 | 2.99 | 7.62 | 1.53 |
|  | Salt Lake County, UT | 13 | 0.60 | 6.70 | 3.95 | 1.94 | 3.29 | 2.07 |
|  | Waukesha County, WI | <10 | 3.30 | 11.10 | 5.90 | 3.00 | 5.42 | 1.55 |
|  |  |  |  |  |  |  |  |  |
|  |  |  |  |  |  |  |  |  |
| **PFNA** |  |  |  |  |  |  |  |  |
| Built in 1981 or After | *Total* | - | *0.30* | *3.90* | *0.91* | *0.61* | *0.77* | *1.72* |
|  | Montgomery County, PA | <10 | 0.40 | 3.60 | 1.43 | 0.97 | 1.18 | 1.96 |
|  | New York City (Queens), NY | 11 | 0.40 | 1.70 | 0.82 | 0.43 | 0.73 | 1.62 |
|  | BYLP, SD/MN | 32 | 0.30 | 1.60 | 0.67 | 0.27 | 0.62 | 1.47 |
|  | Orange County, CA | 11 | 0.60 | 3.90 | 1.26 | 0.99 | 1.05 | 1.78 |
|  | Duplin County, NC | 18 | 0.40 | 2.80 | 1.37 | 0.68 | 1.20 | 1.76 |
|  | Salt Lake County, UT | 42 | 0.30 | 1.60 | 0.72 | 0.33 | 0.65 | 1.57 |
|  | Waukesha County, WI | 22 | 0.30 | 2.90 | 0.88 | 0.66 | 0.75 | 1.71 |
| Built Between 1961-1980 | *Total* | - | *0.20* | *2.10* | *0.80* | *0.42* | *0.70* | *1.66* |
|  | Montgomery County, PA | 11 | 0.60 | 2.10 | 1.35 | 0.45 | 1.28 | 1.46 |
|  | New York City (Queens), NY | <10 | 1.30 | 1.90 | 1.70 | 0.35 | 1.67 | 1.25 |
|  | BYLP, SD/MN | 21 | 0.30 | 1.00 | 0.62 | 0.19 | 0.60 | 1.37 |
|  | Orange County, CA | 15 | 0.20 | 1.60 | 0.75 | 0.39 | 0.65 | 1.78 |
|  | Duplin County, NC | 11 | 0.30 | 1.60 | 0.84 | 0.37 | 0.76 | 1.63 |
|  | Salt Lake County, UT | 31 | 0.30 | 1.40 | 0.65 | 0.30 | 0.60 | 1.52 |
|  | Waukesha County, WI | <10 | 0.40 | 1.10 | 0.66 | 0.32 | 0.60 | 1.60 |
| Built in 1960 or Before | *Total* | - | *0.10* | *4.90* | *1.04* | *0.74* | *0.85* | *1.90* |
|  | Montgomery County, PA | 20 | 0.60 | 2.80 | 1.56 | 0.59 | 1.45 | 1.48 |
|  | New York City (Queens), NY | 13 | 0.40 | 4.90 | 1.45 | 1.37 | 1.06 | 2.17 |
|  | BYLP, SD/MN | 30 | 0.10 | 1.40 | 0.62 | 0.28 | 0.56 | 1.70 |
|  | Orange County, CA | <10 | 0.70 | 1.70 | 1.20 | 0.71 | 1.09 | 1.87 |
|  | Duplin County, NC | <10 | 0.70 | 1.90 | 1.34 | 0.36 | 1.30 | 1.35 |
|  | Salt Lake County, UT | 13 | 0.30 | 1.20 | 0.66 | 0.27 | 0.61 | 1.50 |
|  | Waukesha County, WI | <10 | 0.40 | 1.20 | 0.80 | 0.34 | 0.74 | 1.61 |
|  |  |  |  |  |  |  |  |  |
|  |  |  |  |  |  |  |  |  |
| **PFHxS** |  |  |  |  |  |  |  |  |
| Built in 1981 or After | *Total* | - | *0.07* | *5.30* | *0.75* | *0.70* | *0.55* | *2.24* |
|  | Montgomery County, PA | <10 | 0.07 | 3.10 | 1.35 | 0.93 | 0.95 | 3.06 |
|  | New York City (Queens), NY | 11 | 0.10 | 0.80 | 0.37 | 0.23 | 0.30 | 2.03 |
|  | BYLP, SD/MN | 32 | 0.20 | 5.30 | 1.04 | 1.00 | 0.76 | 2.20 |
|  | Orange County, CA | 11 | 0.40 | 2.40 | 1.01 | 0.66 | 0.85 | 1.82 |
|  | Duplin County, NC | 18 | 0.10 | 1.90 | 0.66 | 0.52 | 0.51 | 2.15 |
|  | Salt Lake County, UT | 42 | 0.07 | 2.00 | 0.55 | 0.43 | 0.43 | 2.07 |
|  | Waukesha County, WI | 22 | 0.07 | 2.30 | 0.62 | 0.44 | 0.51 | 1.95 |
| Built Between 1961-1980 | *Total* | - | *0.07* | *4.50* | *0.82* | *0.80* | *0.59* | *2.18* |
|  | Montgomery County, PA | 11 | 0.07 | 1.70 | 0.90 | 0.54 | 0.69 | 2.49 |
|  | New York City (Queens), NY | <10 | 0.30 | 0.90 | 0.50 | 0.35 | 0.43 | 1.89 |
|  | BYLP, SD/MN | 21 | 0.40 | 4.00 | 1.16 | 0.81 | 0.98 | 1.75 |
|  | Orange County, CA | 15 | 0.20 | 1.30 | 0.58 | 0.34 | 0.50 | 1.76 |
|  | Duplin County, NC | 11 | 0.20 | 1.40 | 0.53 | 0.41 | 0.42 | 2.01 |
|  | Salt Lake County, UT | 31 | 0.20 | 4.50 | 0.82 | 1.10 | 0.50 | 2.39 |
|  | Waukesha County, WI | <10 | 0.30 | 1.80 | 0.84 | 0.62 | 0.68 | 2.08 |
| Built in 1960 or Before | *Total* | - | *0.07* | *3.60* | *0.91* | *0.73* | *0.68* | *2.22* |
|  | Montgomery County, PA | 20 | 0.30 | 3.60 | 1.32 | 0.87 | 1.08 | 1.93 |
|  | New York City (Queens), NY | 13 | 0.20 | 1.30 | 0.43 | 0.35 | 0.34 | 1.94 |
|  | BYLP, SD/MN | 30 | 0.10 | 3.50 | 0.97 | 0.76 | 0.73 | 2.25 |
|  | Orange County, CA | <10 | 0.30 | 0.50 | 0.40 | 0.14 | 0.39 | 1.44 |
|  | Duplin County, NC | <10 | 0.40 | 1.70 | 0.80 | 0.43 | 0.71 | 1.63 |
|  | Salt Lake County, UT | 13 | 0.07 | 2.60 | 0.74 | 0.68 | 0.53 | 2.48 |
|  | Waukesha County, WI | <10 | 0.70 | 2.00 | 1.04 | 0.55 | 0.95 | 1.55 |
|  |  |  |  |  |  |  |  |  |
|  |  |  |  |  |  |  |  |  |
| **PFDA** |  |  |  |  |  |  |  |  |
| Built in 1981 or After | *Total* | - | *0.07* | *1.20* | *0.24* | *0.22* | *0.19* | *2.01* |
|  | Montgomery County, PA | <10 | 0.07 | 0.80 | 0.29 | 0.22 | 0.22 | 2.09 |
|  | New York City (Queens), NY | 11 | 0.07 | 0.80 | 0.29 | 0.23 | 0.22 | 2.35 |
|  | BYLP, SD/MN | 32 | 0.07 | 0.80 | 0.17 | 0.13 | 0.14 | 1.74 |
|  | Orange County, CA | 11 | 0.20 | 1.20 | 0.39 | 0.40 | 0.29 | 2.05 |
|  | Duplin County, NC | 18 | 0.10 | 0.90 | 0.36 | 0.23 | 0.29 | 1.97 |
|  | Salt Lake County, UT | 42 | 0.07 | 0.60 | 0.19 | 0.13 | 0.16 | 1.85 |
|  | Waukesha County, WI | 22 | 0.07 | 1.00 | 0.25 | 0.24 | 0.19 | 2.03 |
| Built Between 1961-1980 | *Total* | - | *0.07* | *1.00* | *0.20* | *0.16* | *0.16* | *1.96* |
|  | Montgomery County, PA | 11 | 0.10 | 0.40 | 0.27 | 0.10 | 0.25 | 1.53 |
|  | New York City (Queens), NY | <10 | 0.60 | 1.00 | 0.77 | 0.21 | 0.75 | 1.30 |
|  | BYLP, SD/MN | 21 | 0.07 | 0.30 | 0.14 | 0.07 | 0.13 | 1.61 |
|  | Orange County, CA | 15 | 0.07 | 0.60 | 0.21 | 0.14 | 0.18 | 1.92 |
|  | Duplin County, NC | 11 | 0.07 | 0.80 | 0.28 | 0.22 | 0.21 | 2.28 |
|  | Salt Lake County, UT | 31 | 0.07 | 0.30 | 0.14 | 0.08 | 0.12 | 1.66 |
|  | Waukesha County, WI | <10 | 0.07 | 0.40 | 0.17 | 0.14 | 0.14 | 2.00 |
| Built in 1960 or Before | *Total* | - | *0.07* | *1.60* | *0.25* | *0.21* | *0.19* | *2.00* |
|  | Montgomery County, PA | 20 | 0.10 | 0.70 | 0.29 | 0.13 | 0.27 | 1.52 |
|  | New York City (Queens), NY | 13 | 0.07 | 1.60 | 0.43 | 0.42 | 0.30 | 2.35 |
|  | BYLP, SD/MN | 30 | 0.07 | 0.40 | 0.14 | 0.08 | 0.12 | 1.68 |
|  | Orange County, CA | <10 | 0.20 | 0.40 | 0.30 | 0.14 | 0.28 | 1.63 |
|  | Duplin County, NC | <10 | 0.10 | 0.60 | 0.37 | 0.17 | 0.33 | 1.75 |
|  | Salt Lake County, UT | 13 | 0.07 | 0.40 | 0.18 | 0.13 | 0.15 | 2.00 |
|  | Waukesha County, WI | <10 | 0.07 | 0.20 | 0.17 | 0.06 | 0.16 | 1.59 |

**Table S8.** Summary statistics for serum PFAS measurements (ng/mL) for the NCS IVS cohort (Total) and the 7 NCS IVS counties, subdivided by self-reported drinking water source. Total number of responses for each response category is censored to preserve participant confidentiality.

|  |  | N | Min | Max | AM | SD | GM | GMSD |
| --- | --- | --- | --- | --- | --- | --- | --- | --- |
| **PFOA** |  |  |  |  |  |  |  |  |
| Tap Water | *Total* | - | *0.30* | *4.60* | *1.57* | *0.91* | *1.32* | *1.84* |
|  | Montgomery County, PA | <10 | 1.20 | 4.60 | 2.29 | 1.23 | 2.06 | 1.60 |
|  | New York City (Queens), NY | <10 | 0.30 | 2.20 | 1.10 | 0.70 | 0.89 | 2.08 |
|  | BYLP, SD/MN | 31 | 0.40 | 3.40 | 1.38 | 0.74 | 1.19 | 1.78 |
|  | Orange County, CA | <10 | 0.80 | 3.00 | 2.08 | 0.88 | 1.89 | 1.71 |
|  | Duplin County, NC | 26 | 0.70 | 4.20 | 1.85 | 0.97 | 1.61 | 1.72 |
|  | Salt Lake County, UT | 35 | 0.40 | 3.60 | 1.24 | 0.74 | 1.06 | 1.76 |
|  | Waukesha County, WI | 11 | 0.70 | 4.00 | 2.10 | 0.96 | 1.89 | 1.64 |
| Filtered Tap Water | *Total* | - | *0.40* | *7.20* | *1.85* | *1.27* | *1.53* | *1.83* |
|  | Montgomery County, PA | 26 | 0.50 | 6.70 | 2.36 | 1.42 | 1.97 | 1.90 |
|  | New York City (Queens), NY | 13 | 0.40 | 5.60 | 1.82 | 1.51 | 1.40 | 2.09 |
|  | BYLP, SD/MN | 46 | 0.60 | 6.40 | 1.65 | 1.03 | 1.44 | 1.65 |
|  | Orange County, CA | 19 | 0.40 | 6.60 | 2.14 | 1.44 | 1.76 | 1.94 |
|  | Duplin County, NC | 10 | 0.70 | 3.70 | 2.17 | 0.93 | 1.96 | 1.67 |
|  | Salt Lake County, UT | 38 | 0.50 | 4.30 | 1.50 | 0.82 | 1.33 | 1.64 |
|  | Waukesha County, WI | 13 | 0.50 | 7.20 | 1.95 | 2.11 | 1.33 | 2.34 |
| Bottled Water | *Total* | - | *0.20* | *6.90* | *1.45* | *0.97* | *1.23* | *1.77* |
|  | Montgomery County, PA | 12 | 1.00 | 4.10 | 2.23 | 0.93 | 2.06 | 1.52 |
|  | New York City (Queens), NY | 12 | 0.30 | 6.90 | 1.72 | 1.73 | 1.29 | 2.10 |
|  | BYLP, SD/MN | 12 | 0.50 | 1.80 | 1.12 | 0.39 | 1.05 | 1.45 |
|  | Orange County, CA | 24 | 0.50 | 3.40 | 1.23 | 0.63 | 1.11 | 1.57 |
|  | Duplin County, NC | 31 | 0.20 | 6.10 | 1.52 | 1.03 | 1.29 | 1.80 |
|  | Salt Lake County, UT | 17 | 0.40 | 2.70 | 1.30 | 0.63 | 1.15 | 1.70 |
|  | Waukesha County, WI | <10 | 0.30 | 2.00 | 1.00 | 0.64 | 0.81 | 2.07 |
|  |  |  |  |  |  |  |  |  |
|  |  |  |  |  |  |  |  |  |
| **PFOS** |  |  |  |  |  |  |  |  |
| Tap Water | *Total* | - | *0.60* | *27.10* | *4.84* | *3.59* | *3.92* | *1.93* |
|  | Montgomery County, PA | <10 | 3.20 | 11.50 | 5.45 | 2.61 | 5.05 | 1.49 |
|  | New York City (Queens), NY | <10 | 0.60 | 27.10 | 7.19 | 9.26 | 3.77 | 3.28 |
|  | BYLP, SD/MN | 31 | 1.10 | 9.70 | 4.40 | 2.07 | 3.89 | 1.70 |
|  | Orange County, CA | <10 | 2.40 | 10.40 | 6.48 | 3.03 | 5.80 | 1.76 |
|  | Duplin County, NC | 26 | 1.60 | 14.70 | 6.16 | 3.48 | 5.29 | 1.77 |
|  | Salt Lake County, UT | 35 | 0.60 | 6.70 | 3.09 | 1.69 | 2.67 | 1.76 |
|  | Waukesha County, WI | 11 | 1.60 | 11.10 | 5.43 | 2.76 | 4.83 | 1.69 |
| Filtered Tap Water | *Total* | - | *0.30* | *14.70* | *4.63* | *2.71* | *3.89* | *1.86* |
|  | Montgomery County, PA | 26 | 1.00 | 12.70 | 4.55 | 3.15 | 3.58 | 2.08 |
|  | New York City (Queens), NY | 13 | 1.40 | 14.70 | 4.14 | 3.83 | 3.06 | 2.16 |
|  | BYLP, SD/MN | 46 | 0.30 | 12.70 | 5.07 | 2.49 | 4.36 | 1.89 |
|  | Orange County, CA | 19 | 1.30 | 13.60 | 4.66 | 3.09 | 3.93 | 1.79 |
|  | Duplin County, NC | 10 | 2.00 | 10.20 | 6.92 | 2.70 | 6.27 | 1.68 |
|  | Salt Lake County, UT | 38 | 1.10 | 9.60 | 3.91 | 1.70 | 3.57 | 1.55 |
|  | Waukesha County, WI | 13 | 1.20 | 10.20 | 3.98 | 2.39 | 3.38 | 1.83 |
| Bottled Water | *Total* | - | *0.14* | *208.00* | *7.11* | *20.28* | *3.87* | *2.42* |
|  | Montgomery County, PA | 12 | 1.50 | 15.90 | 6.41 | 4.16 | 5.29 | 1.94 |
|  | New York City (Queens), NY | 12 | 0.90 | 14.10 | 4.57 | 3.56 | 3.56 | 2.12 |
|  | BYLP, SD/MN | 12 | 0.90 | 10.40 | 4.11 | 2.43 | 3.48 | 1.89 |
|  | Orange County, CA | 24 | 0.90 | 7.30 | 3.05 | 1.47 | 2.69 | 1.71 |
|  | Duplin County, NC | 31 | 0.14 | 208.00 | 15.48 | 37.98 | 5.91 | 3.51 |
|  | Salt Lake County, UT | 17 | 0.90 | 7.10 | 3.35 | 1.95 | 2.84 | 1.84 |
|  | Waukesha County, WI | <10 | 1.20 | 7.10 | 3.90 | 1.89 | 3.45 | 1.77 |
|  |  |  |  |  |  |  |  |  |
|  |  |  |  |  |  |  |  |  |
| **PFNA** |  |  |  |  |  |  |  |  |
| Tap Water | *Total* | - | *0.20* | *6.80* | *0.95* | *0.83* | *0.77* | *1.84* |
|  | Montgomery County, PA | <10 | 0.90 | 2.60 | 1.66 | 0.56 | 1.58 | 1.40 |
|  | New York City (Queens), NY | <10 | 0.20 | 6.80 | 1.79 | 2.36 | 0.94 | 3.13 |
|  | BYLP, SD/MN | 31 | 0.20 | 1.40 | 0.62 | 0.26 | 0.57 | 1.52 |
|  | Orange County, CA | <10 | 0.90 | 1.10 | 0.98 | 0.08 | 0.98 | 1.09 |
|  | Duplin County, NC | 26 | 0.50 | 2.80 | 1.23 | 0.59 | 1.10 | 1.59 |
|  | Salt Lake County, UT | 35 | 0.30 | 1.40 | 0.65 | 0.32 | 0.58 | 1.62 |
|  | Waukesha County, WI | 11 | 0.40 | 2.90 | 0.98 | 0.67 | 0.85 | 1.67 |
| Filtered Tap Water | *Total* | - | *0.10* | *3.90* | *0.92* | *0.63* | *0.78* | *1.75* |
|  | Montgomery County, PA | 26 | 0.40 | 3.60 | 1.45 | 0.75 | 1.28 | 1.70 |
|  | New York City (Queens), NY | 13 | 0.40 | 3.60 | 1.05 | 0.85 | 0.86 | 1.82 |
|  | BYLP, SD/MN | 46 | 0.10 | 1.60 | 0.65 | 0.26 | 0.59 | 1.56 |
|  | Orange County, CA | 19 | 0.20 | 3.90 | 1.08 | 0.83 | 0.88 | 1.89 |
|  | Duplin County, NC | 10 | 0.40 | 2.20 | 1.31 | 0.60 | 1.16 | 1.77 |
|  | Salt Lake County, UT | 38 | 0.30 | 1.60 | 0.71 | 0.30 | 0.66 | 1.44 |
|  | Waukesha County, WI | 13 | 0.40 | 2.70 | 0.81 | 0.61 | 0.69 | 1.71 |
| Bottled Water | *Total* | - | *0.20* | *3.30* | *0.88* | *0.51* | *0.76* | *1.73* |
|  | Montgomery County, PA | 12 | 0.80 | 1.90 | 1.28 | 0.34 | 1.24 | 1.31 |
|  | New York City (Queens), NY | 12 | 0.40 | 3.30 | 1.21 | 0.87 | 0.97 | 1.98 |
|  | BYLP, SD/MN | 12 | 0.30 | 1.00 | 0.63 | 0.23 | 0.60 | 1.44 |
|  | Orange County, CA | 24 | 0.20 | 1.60 | 0.64 | 0.27 | 0.59 | 1.51 |
|  | Duplin County, NC | 31 | 0.20 | 2.70 | 1.12 | 0.51 | 0.99 | 1.71 |
|  | Salt Lake County, UT | 17 | 0.30 | 1.20 | 0.62 | 0.24 | 0.58 | 1.47 |
|  | Waukesha County, WI | <10 | 0.30 | 0.70 | 0.49 | 0.16 | 0.46 | 1.41 |
|  |  |  |  |  |  |  |  |  |
|  |  |  |  |  |  |  |  |  |
| **PFHxS** |  |  |  |  |  |  |  |  |
| Tap Water | *Total* | - | *0.07* | *3.60* | *0.74* | *0.63* | *0.54* | *2.27* |
|  | Montgomery County, PA | <10 | 0.50 | 3.60 | 1.43 | 0.92 | 1.24 | 1.72 |
|  | New York City (Queens), NY | <10 | 0.07 | 1.40 | 0.43 | 0.42 | 0.31 | 2.38 |
|  | BYLP, SD/MN | 31 | 0.07 | 3.50 | 0.91 | 0.72 | 0.69 | 2.22 |
|  | Orange County, CA | <10 | 0.30 | 1.90 | 1.00 | 0.64 | 0.82 | 2.10 |
|  | Duplin County, NC | 26 | 0.20 | 2.10 | 0.75 | 0.55 | 0.58 | 2.05 |
|  | Salt Lake County, UT | 35 | 0.07 | 2.60 | 0.50 | 0.47 | 0.38 | 2.07 |
|  | Waukesha County, WI | 11 | 0.07 | 1.80 | 0.69 | 0.46 | 0.54 | 2.32 |
| Filtered Tap Water | *Total* | - | *0.07* | *5.30* | *0.91* | *0.90* | *0.63* | *2.38* |
|  | Montgomery County, PA | 26 | 0.07 | 4.90 | 1.23 | 1.10 | 0.82 | 2.76 |
|  | New York City (Queens), NY | 13 | 0.10 | 0.50 | 0.22 | 0.12 | 0.19 | 1.72 |
|  | BYLP, SD/MN | 46 | 0.10 | 5.30 | 1.17 | 0.99 | 0.88 | 2.19 |
|  | Orange County, CA | 19 | 0.30 | 2.40 | 0.76 | 0.51 | 0.65 | 1.72 |
|  | Duplin County, NC | 10 | 0.20 | 1.30 | 0.52 | 0.33 | 0.45 | 1.74 |
|  | Salt Lake County, UT | 38 | 0.10 | 4.50 | 0.84 | 1.00 | 0.55 | 2.38 |
|  | Waukesha County, WI | 13 | 0.20 | 2.00 | 0.75 | 0.46 | 0.64 | 1.84 |
| Bottled Water | *Total* | - | *0.07* | *12.60* | *0.76* | *1.27* | *0.50* | *2.25* |
|  | Montgomery County, PA | 12 | 0.30 | 4.70 | 1.41 | 1.23 | 1.05 | 2.21 |
|  | New York City (Queens), NY | 12 | 0.07 | 1.30 | 0.44 | 0.36 | 0.32 | 2.37 |
|  | BYLP, SD/MN | 12 | 0.10 | 2.20 | 0.78 | 0.63 | 0.58 | 2.27 |
|  | Orange County, CA | 24 | 0.20 | 1.50 | 0.59 | 0.29 | 0.53 | 1.61 |
|  | Duplin County, NC | 31 | 0.10 | 12.60 | 0.90 | 2.20 | 0.46 | 2.41 |
|  | Salt Lake County, UT | 17 | 0.10 | 2.00 | 0.52 | 0.47 | 0.38 | 2.20 |
|  | Waukesha County, WI | <10 | 0.30 | 2.30 | 0.69 | 0.72 | 0.51 | 2.08 |
|  |  |  |  |  |  |  |  |  |
|  |  |  |  |  |  |  |  |  |
| **PFDA** |  |  |  |  |  |  |  |  |
| Tap Water | *Total* | - | *0.07* | *2.50* | *0.25* | *0.29* | *0.18* | *2.11* |
|  | Montgomery County, PA | <10 | 0.20 | 0.50 | 0.30 | 0.11 | 0.28 | 1.41 |
|  | New York City (Queens), NY | <10 | 0.07 | 2.50 | 0.66 | 0.85 | 0.30 | 3.92 |
|  | BYLP, SD/MN | 31 | 0.07 | 0.40 | 0.15 | 0.08 | 0.13 | 1.65 |
|  | Orange County, CA | <10 | 0.20 | 0.30 | 0.24 | 0.05 | 0.24 | 1.25 |
|  | Duplin County, NC | 26 | 0.07 | 0.90 | 0.33 | 0.21 | 0.27 | 1.93 |
|  | Salt Lake County, UT | 35 | 0.07 | 0.50 | 0.16 | 0.11 | 0.13 | 1.85 |
|  | Waukesha County, WI | 11 | 0.07 | 0.90 | 0.24 | 0.23 | 0.19 | 2.04 |
| Filtered Tap Water | *Total* | - | *0.07* | *1.20* | *0.23* | *0.19* | *0.18* | *1.95* |
|  | Montgomery County, PA | 26 | 0.07 | 0.80 | 0.29 | 0.16 | 0.25 | 1.72 |
|  | New York City (Queens), NY | 13 | 0.07 | 0.90 | 0.29 | 0.23 | 0.22 | 2.12 |
|  | BYLP, SD/MN | 46 | 0.07 | 0.80 | 0.16 | 0.12 | 0.14 | 1.73 |
|  | Orange County, CA | 19 | 0.07 | 1.20 | 0.32 | 0.32 | 0.23 | 2.10 |
|  | Duplin County, NC | 10 | 0.07 | 0.70 | 0.40 | 0.18 | 0.35 | 1.88 |
|  | Salt Lake County, UT | 38 | 0.07 | 0.60 | 0.18 | 0.11 | 0.16 | 1.75 |
|  | Waukesha County, WI | 13 | 0.07 | 1.00 | 0.24 | 0.25 | 0.17 | 2.23 |
| Bottled Water | *Total* | - | *0.07* | *2.60* | *0.26* | *0.30* | *0.18* | *2.16* |
|  | Montgomery County, PA | 12 | 0.10 | 0.40 | 0.23 | 0.12 | 0.20 | 1.76 |
|  | New York City (Queens), NY | 12 | 0.10 | 1.00 | 0.39 | 0.31 | 0.28 | 2.42 |
|  | BYLP, SD/MN | 12 | 0.07 | 0.20 | 0.10 | 0.05 | 0.10 | 1.46 |
|  | Orange County, CA | 24 | 0.07 | 0.60 | 0.18 | 0.11 | 0.16 | 1.73 |
|  | Duplin County, NC | 31 | 0.07 | 2.60 | 0.43 | 0.47 | 0.30 | 2.34 |
|  | Salt Lake County, UT | 17 | 0.07 | 0.40 | 0.15 | 0.09 | 0.13 | 1.71 |
|  | Waukesha County, WI | <10 | 0.07 | 0.20 | 0.12 | 0.06 | 0.11 | 1.59 |

**Table S9.** P-values for pairwise t-tests between serum PFOA concentrations for the same drinking water source response groups in each NCS IVS county, adjusted for multiple comparisons. Asterisk indicates statistical significance at *p*<0.05.

| *Tap Water* |  |  |  |  |  |  |
| --- | --- | --- | --- | --- | --- | --- |
|  | Duplin | Montgomery | Orange | Queens | Salt Lake | Waukesha |
| BYLP | 0.4165 | 0.1896 | 0.6218 | 0.8153 | 0.9821 | 0.2324 |
| Duplin | - | 0.9332 | 0.9973 | 0.1016 | 0.0752 | 0.9842 |
| Montgomery | - | - | 1.0000 | 0.0418* | 0.0511 | 0.9999 |
| Orange | - | - | - | 0.2101 | 0.3413 | 1.0000 |
| Queens | - | - | - | - | 0.9793 | 0.0519 |
| Salt Lake | - | - | - | - | - | 0.0546 |
|  |  |  |  |  |  |  |
| *Filtered Tap Water* | | |  |  |  |  |
|  | Duplin | Montgomery | Orange | Queens | Salt Lake | Waukesha |
| BYLP | 0.7547 | 0.3249 | 0.8775 | 1.0000 | 0.9958 | 0.9996 |
| Duplin | - | 1.0000 | 0.9993 | 0.8259 | 0.5213 | 0.7245 |
| Montgomery | - | - | 0.9956 | 0.6106 | 0.1268 | 0.4614 |
| Orange | - | - | - | 0.9325 | 0.6216 | 0.8541 |
| Queens | - | - | - | - | 1.0000 | 1.0000 |
| Salt Lake | - | - | - | - | - | 1.0000 |
|  |  |  |  |  |  |  |
| *Bottled Water* |  |  |  |  |  |  |
|  | Duplin | Montgomery | Orange | Queens | Salt Lake | Waukesha |
| BYLP | 0.9309 | 0.0504 | 1.0000 | 0.9705 | 0.9995 | 0.9532 |
| Duplin | - | 0.1631 | 0.9540 | 1.0000 | 0.9934 | 0.4095 |
| Montgomery | - | - | 0.0301* | 0.3639 | 0.0816 | 0.0092* |
| Orange | - | - | - | 0.9873 | 1.0000 | 0.8335 |
| Queens | - | - | - | - | 0.9980 | 0.5651 |
| Salt Lake | - | - | - | - | - | 0.7883 |

**Table S10.** Summary statistics for serum PFAS measurements (ng/mL) for the NCS IVS cohort (Total) and the 7 NCS IVS counties, subdivided by self-reported quantity of French fries eaten per sitting. Total number of responses for each response category is censored to preserve participant confidentiality.

|  |  | N | Min | Max | AM | SD | GM | GMSD |
| --- | --- | --- | --- | --- | --- | --- | --- | --- |
| **PFOA** |  |  |  |  |  |  |  |  |
| Less than 10 or less than 1/2 cup | *Total* | - | *0.30* | *6.60* | *1.50* | *1.10* | *1.23* | *1.87* |
|  | Montgomery County, PA | <10 | 1.10 | 4.10 | 2.36 | 1.09 | 2.15 | 1.59 |
|  | New York City (Queens), NY | <10 | 0.30 | 2.20 | 0.97 | 0.58 | 0.82 | 1.86 |
|  | BYLP, SD/MN | 16 | 0.40 | 6.40 | 1.38 | 1.39 | 1.09 | 1.84 |
|  | Orange County, CA | 12 | 0.50 | 6.60 | 1.87 | 1.69 | 1.42 | 2.08 |
|  | Duplin County, NC | 14 | 0.70 | 4.20 | 1.61 | 0.87 | 1.45 | 1.60 |
|  | Salt Lake County, UT | 29 | 0.40 | 3.40 | 1.39 | 0.79 | 1.20 | 1.75 |
|  | Waukesha County, WI | <10 | 0.30 | 1.80 | 1.09 | 0.65 | 0.87 | 2.16 |
| 10 or more or  1/2 cup or more | *Total* | - | *0.20* | *7.20* | *1.76* | *1.16* | *1.46* | *1.84* |
|  | Montgomery County, PA | 35 | 0.50 | 6.70 | 2.36 | 1.34 | 2.01 | 1.81 |
|  | New York City (Queens), NY | 14 | 0.30 | 6.90 | 2.23 | 1.90 | 1.66 | 2.23 |
|  | BYLP, SD/MN | 69 | 0.40 | 4.00 | 1.51 | 0.70 | 1.36 | 1.62 |
|  | Orange County, CA | 17 | 0.40 | 4.70 | 1.95 | 1.25 | 1.56 | 2.09 |
|  | Duplin County, NC | 38 | 0.20 | 6.10 | 1.78 | 1.10 | 1.49 | 1.90 |
|  | Salt Lake County, UT | 52 | 0.40 | 4.30 | 1.39 | 0.84 | 1.19 | 1.77 |
|  | Waukesha County, WI | 23 | 0.50 | 7.20 | 1.91 | 1.62 | 1.52 | 1.91 |
|  |  |  |  |  |  |  |  |  |
|  |  |  |  |  |  |  |  |  |
| **PFOS** |  |  |  |  |  |  |  |  |
| Less than 10 or less than 1/2 cup | *Total* | - | *0.30* | *208.00* | *6.40* | *21.29* | *3.37* | *2.34* |
|  | Montgomery County, PA | <10 | 1.90 | 12.70 | 5.89 | 4.04 | 4.85 | 1.93 |
|  | New York City (Queens), NY | <10 | 0.60 | 3.70 | 2.01 | 0.92 | 1.80 | 1.70 |
|  | BYLP, SD/MN | 16 | 0.30 | 7.00 | 3.47 | 1.81 | 2.82 | 2.21 |
|  | Orange County, CA | 12 | 0.90 | 7.20 | 3.26 | 1.93 | 2.71 | 1.96 |
|  | Duplin County, NC | 14 | 1.60 | 208.00 | 22.46 | 53.98 | 7.55 | 3.46 |
|  | Salt Lake County, UT | 29 | 0.90 | 13.80 | 3.81 | 2.72 | 3.10 | 1.92 |
|  | Waukesha County, WI | <10 | 1.20 | 5.70 | 3.36 | 1.52 | 3.03 | 1.68 |
| 10 or more or  1/2 cup or more | *Total* | - | *0.14* | *27.10* | *4.98* | *3.25* | *4.12* | *1.90* |
|  | Montgomery County, PA | 35 | 1.00 | 15.90 | 5.30 | 3.28 | 4.34 | 1.97 |
|  | New York City (Queens), NY | 14 | 1.40 | 27.10 | 6.94 | 7.12 | 4.83 | 2.33 |
|  | BYLP, SD/MN | 69 | 0.90 | 12.70 | 4.97 | 2.34 | 4.43 | 1.66 |
|  | Orange County, CA | 17 | 1.30 | 13.60 | 4.62 | 3.01 | 3.89 | 1.82 |
|  | Duplin County, NC | 38 | 0.14 | 16.90 | 6.19 | 3.84 | 4.90 | 2.29 |
|  | Salt Lake County, UT | 52 | 0.60 | 7.40 | 3.48 | 1.74 | 3.05 | 1.71 |
|  | Waukesha County, WI | 23 | 1.20 | 11.10 | 4.92 | 2.55 | 4.33 | 1.71 |
|  |  |  |  |  |  |  |  |  |
|  |  |  |  |  |  |  |  |  |
| **PFNA** |  |  |  |  |  |  |  |  |
| Less than 10 or less than 1/2 cup | *Total* | - | *0.10* | *3.90* | *0.84* | *0.61* | *0.69* | *1.85* |
|  | Montgomery County, PA | <10 | 0.60 | 2.30 | 1.29 | 0.52 | 1.20 | 1.51 |
|  | New York City (Queens), NY | <10 | 0.20 | 1.20 | 0.68 | 0.32 | 0.60 | 1.73 |
|  | BYLP, SD/MN | 16 | 0.10 | 1.60 | 0.55 | 0.31 | 0.48 | 1.74 |
|  | Orange County, CA | 12 | 0.20 | 3.90 | 0.95 | 0.97 | 0.71 | 2.10 |
|  | Duplin County, NC | 14 | 0.70 | 2.80 | 1.46 | 0.69 | 1.33 | 1.55 |
|  | Salt Lake County, UT | 29 | 0.30 | 1.60 | 0.67 | 0.33 | 0.60 | 1.59 |
|  | Waukesha County, WI | <10 | 0.30 | 0.70 | 0.50 | 0.17 | 0.47 | 1.44 |
| 10 or more or  1/2 cup or more | *Total* | - | *0.20* | *6.80* | *0.95* | *0.68* | *0.80* | *1.75* |
|  | Montgomery County, PA | 35 | 0.40 | 3.60 | 1.49 | 0.68 | 1.35 | 1.59 |
|  | New York City (Queens), NY | 14 | 0.40 | 6.80 | 1.76 | 1.78 | 1.22 | 2.35 |
|  | BYLP, SD/MN | 69 | 0.20 | 1.40 | 0.66 | 0.23 | 0.62 | 1.46 |
|  | Orange County, CA | 17 | 0.20 | 2.00 | 0.96 | 0.50 | 0.83 | 1.81 |
|  | Duplin County, NC | 38 | 0.20 | 2.00 | 1.11 | 0.48 | 0.99 | 1.69 |
|  | Salt Lake County, UT | 52 | 0.30 | 1.50 | 0.68 | 0.30 | 0.62 | 1.53 |
|  | Waukesha County, WI | 23 | 0.40 | 2.70 | 0.86 | 0.47 | 0.78 | 1.52 |
|  |  |  |  |  |  |  |  |  |
|  |  |  |  |  |  |  |  |  |
| **PFHxS** |  |  |  |  |  |  |  |  |
| Less than 10 or less than 1/2 cup | *Total* | - | *0.07* | *3.90* | *0.70* | *0.75* | *0.45* | *2.52* |
|  | Montgomery County, PA | <10 | 0.40 | 3.60 | 1.39 | 1.06 | 1.09 | 2.10 |
|  | New York City (Queens), NY | <10 | 0.07 | 0.20 | 0.14 | 0.06 | 0.13 | 1.52 |
|  | BYLP, SD/MN | 16 | 0.07 | 2.20 | 0.93 | 0.65 | 0.64 | 2.83 |
|  | Orange County, CA | 12 | 0.20 | 2.40 | 0.66 | 0.66 | 0.49 | 2.09 |
|  | Duplin County, NC | 14 | 0.20 | 1.10 | 0.51 | 0.31 | 0.43 | 1.80 |
|  | Salt Lake County, UT | 29 | 0.07 | 3.90 | 0.74 | 0.91 | 0.45 | 2.59 |
|  | Waukesha County, WI | <10 | 0.30 | 0.60 | 0.37 | 0.11 | 0.36 | 1.30 |
| 10 or more or  1/2 cup or more | *Total* | - | *0.07* | *12.60* | *0.92* | *1.11* | *0.64* | *2.27* |
|  | Montgomery County, PA | 35 | 0.07 | 4.90 | 1.35 | 1.13 | 0.96 | 2.55 |
|  | New York City (Queens), NY | 14 | 0.07 | 1.40 | 0.53 | 0.42 | 0.39 | 2.29 |
|  | BYLP, SD/MN | 69 | 0.10 | 5.30 | 1.06 | 0.90 | 0.82 | 2.03 |
|  | Orange County, CA | 17 | 0.30 | 2.40 | 0.78 | 0.61 | 0.63 | 1.91 |
|  | Duplin County, NC | 38 | 0.10 | 12.60 | 0.93 | 2.00 | 0.51 | 2.43 |
|  | Salt Lake County, UT | 52 | 0.07 | 4.50 | 0.65 | 0.72 | 0.47 | 2.16 |
|  | Waukesha County, WI | 23 | 0.20 | 2.30 | 0.81 | 0.54 | 0.68 | 1.82 |
|  |  |  |  |  |  |  |  |  |
|  |  |  |  |  |  |  |  |  |
| **PFDA** |  |  |  |  |  |  |  |  |
| Less than 10 or less than 1/2 cup | *Total* | - | *0.07* | *2.60* | *0.25* | *0.33* | *0.17* | *2.21* |
|  | Montgomery County, PA | <10 | 0.10 | 0.40 | 0.24 | 0.12 | 0.21 | 1.72 |
|  | New York City (Queens), NY | <10 | 0.07 | 0.80 | 0.22 | 0.24 | 0.15 | 2.35 |
|  | BYLP, SD/MN | 16 | 0.07 | 0.80 | 0.15 | 0.18 | 0.11 | 1.87 |
|  | Orange County, CA | 12 | 0.07 | 1.20 | 0.27 | 0.31 | 0.19 | 2.21 |
|  | Duplin County, NC | 14 | 0.20 | 2.60 | 0.61 | 0.64 | 0.44 | 2.18 |
|  | Salt Lake County, UT | 29 | 0.07 | 0.50 | 0.17 | 0.12 | 0.14 | 1.82 |
|  | Waukesha County, WI | <10 | 0.07 | 0.30 | 0.13 | 0.09 | 0.11 | 1.77 |
| 10 or more or  1/2 cup or more | *Total* | - | *0.07* | *2.50* | *0.24* | *0.23* | *0.18* | *2.01* |
|  | Montgomery County, PA | 35 | 0.07 | 0.80 | 0.28 | 0.16 | 0.25 | 1.72 |
|  | New York City (Queens), NY | 14 | 0.10 | 2.50 | 0.58 | 0.63 | 0.39 | 2.50 |
|  | BYLP, SD/MN | 69 | 0.07 | 0.40 | 0.15 | 0.08 | 0.14 | 1.63 |
|  | Orange County, CA | 17 | 0.07 | 1.20 | 0.32 | 0.28 | 0.24 | 2.11 |
|  | Duplin County, NC | 38 | 0.07 | 0.70 | 0.32 | 0.19 | 0.26 | 2.01 |
|  | Salt Lake County, UT | 52 | 0.07 | 0.60 | 0.16 | 0.12 | 0.13 | 1.84 |
|  | Waukesha County, WI | 23 | 0.07 | 1.00 | 0.22 | 0.19 | 0.18 | 1.86 |
